# Differential metabolic and cellular brain regional susceptibility in adult rats with chronic liver disease

**DOI:** 10.1007/s11011-026-01954-1

**Published:** 2026-09-11

**Authors:** Dunja Simicic, Katarzyna Pierzchala, Olivier Braissant, Dario Sessa, Valérie McLin, Cristina Cudalbu

**Affiliations:** 1 CIBM Center for Biomedical Imaging, Lausanne, Switzerland; 2 CIBM Pre-Clinical Imaging Section, École polytechnique fédérale de Lausanne (EPFL), Lausanne, Switzerland; 3 Service of Clinical Chemistry, University of Lausanne and Lausanne University Hospital, Lausanne, Switzerland; 4 Swiss Pediatric Liver Center, Pediatric Gastroenterology, Hepatology and Nutrition Unit, Department of Pediatrics, Gynecology and Obstetrics, University of Geneva, Geneva, Switzerland; 5 MRS4Brain group, School of Basic Sciences, École Polytechnique Fédérale de Lausanne (EPFL), Lausanne, Switzerland

**Keywords:** Proton magnetic resonance spectroscopy, Bile duct ligation, Type C hepatic encephalopathy, Glutamine, Central nervous system

## Abstract

Patients with type C hepatic encephalopathy (HE) present diverse symptoms, indicating disease-associated effects in multiple brain regions. While identifying the distinct metabolic and cellular changes in these regions could help explain the clinical variability of HE, this remains challenging. By leveraging the non-invasive capabilities of in-vivo ^1^H-MR Spectroscopy (9.4T) and complementary ex-vivo histological assessments, we analyzed, longitudinal neurometabolic and morphological changes in the hippocampus, striatum, and cerebellum during the progression of chronic liver disease, induced type C HE in the bile duct ligated rat model (*n* = 41). We showed that while brain regions share common metabolic and cellular responses such as acute glutamine increase, glial activation, and neuronal alterations, they also display unique characteristics. The most striking difference was in the cerebellum, where the highest glutamine load (+ 134%) was associated with elevated lactate and reduced Purkinje soma surface, while the striatum showed the strongest osmolyte decrease despite having the lowest glutamine increase among the studied regions. Based on the early rise of glutamine across regions, we highlight glutamine as one of the molecular drivers of a cascade of metabolic and cellular abnormalities, together with ^1^H-MRS as a valuable modality to investigate region-specific brain alterations in HE both in animal models and humans.

**Graphical abstract:**

**Figure:**
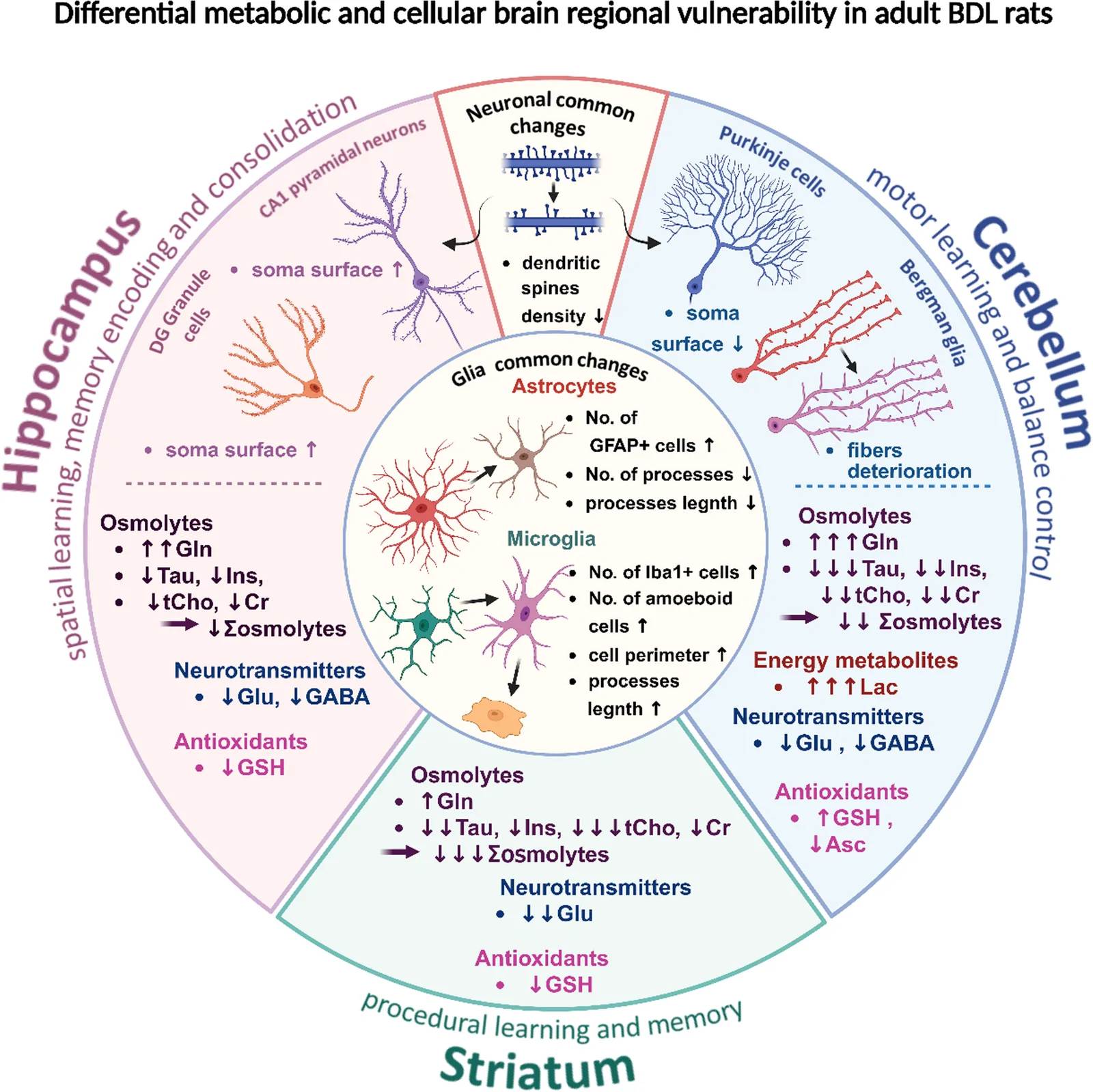

**Supplementary Information:**

The online version contains supplementary material available at https://doi.org/10.1007/s11011-026-01954-1.

## Introduction

Patients with type C hepatic encephalopathy (HE) present a wide spectrum of symptoms including motor and cognition impairment, suggesting that different brain regions may be involved and underlie the varying phenotypes (Cudalbu and Taylor-Robinson 2019). Although the hippocampus, cerebellum, and striatum have been identified as key regions implicated in HE in rat models and human subjects (Cudalbu and Taylor-Robinson 2019), little is known about the metabolic and cellular differences across these regions, something that would help understand the breadth of the clinical spectrum.

The hippocampus supports spatial cognition, memory and learning, which can be impaired in cirrhotic patients by altered cellular morphology, neuroinflammation (Pierzchala et al. 2022) or disrupted structural and functional connectivity (Ahl et al. 2004; Balzano et al. 2018; García-García et al. 2018; Pierzchala et al. 2022). The cerebellum contributes to motor ability, spatial navigation and working memory (Ackerman 1992). Studies in type C HE models suggested that neuroinflammation might affect the cerebellum earlier than other brain regions (Pierzchala et al. 2022), potentially contributing to cognitive and motor impairments. In patients with varying liver disease severity, cerebellar inflammation occurred early, even before cirrhosis (Balzano et al. 2018). The striatum, involved in motor control, reward, and executive systems (Ackerman 1992), contributed to disturbed thalamostriatal connectivity in cirrhotic patients, implicating this system in HE-related cognitive deficits (García-García et al. 2018). While brain regional differences contribute to type C HE and its neurological symptoms, non-invasive tools to probe these differences remain limited.

Diagnosing HE in patients and animal models involves sophisticated neuropsychometric, neurophysiological, and neurobehavioral tests (Bémeur et al. 2016; Häussinger et al. 2022). However, early or mild neurological alterations are often difficult to detect. Simple sensory, motor, and cognitive tasks require coordination across multiple brain regions (Schnitzler and Gross 2005), and HE is associated with diverse symptoms affecting different subsystems (Butz et al. 2013). The mechanisms behind these neurological impairments remain unclear, highlighting the need for further studies to identify the specific brain areas and neurochemical changes involved. In this context, proton magnetic resonance spectroscopy (^1^H-MRS) enables noninvasive, in-vivo measurements of brain metabolites, allowing potential region-specific alterations to be linked to the clinical spectrum observed in patients.

Few ^1^H-MRS studies in patients with type C HE evaluated brain regional differences in metabolic ratios of tCho (total choline), Glx (glutamine+glutamate) and NAA (N-acetylaspartate) to creatine (Cr) (Taylor-Robinson et al. 1994), showing lower Cho/Cr in the occipital cortex and higher Glx/Cr in the basal ganglia. These studies have been performed at a low magnetic field, thus limiting the content of metabolic information. At the cellular level, astrocytes were shown as implicated in HE. While emerging evidence points to the involvement of additional cell types (neurons and microglia), comprehensive investigations of their specific roles remain limited (Angelova et al. 2022; Pierzchala et al. 2024; Mosso et al. 2024). Analyzing different brain regions at both the cellular and metabolic levels in a type C HE model may therefore provide insights into the mechanisms underlying the complexity of HE^8^. In the bile duct-ligated (BDL) rat model, we have previously shown distinct glutamine (Gln) alterations between the hippocampus and cerebellum together with slight differences in oxidative stress and neuroinflammation (Pierzchala et al. 2022), supporting the need for a more thorough regional brain exploration in type C HE.

Therefore, our aim was to analyze the longitudinal in-vivo neurometabolic and morphological changes of astrocytes, neurons and microglia in hippocampus, striatum and cerebellum during the progression of chronic liver disease (CLD)-induced type C HE in the BDL rat model, using a multimodal approach combining ^1^H-MRS with histology and blood biochemistry. Our objective was to: (1) identify common and differential neurometabolic and cellular patterns across brain structures in a model of type C HE, and (2) pinpoint potential local or systemic drivers of disease.

## Materials and methods

### BDL rat model of CLD induced-type C HE

All experiments were approved by the Committee on Animal Experimentation for the Canton de Vaud, Switzerland (VD3022/VD2439) and were conformed to ARRIVE guidelines. Wistar male rats (*n* = 41, 175–200 g, Charles River Laboratories, L’Arbresle, France) were used: 32 rats underwent BDL surgery (DeMorrow et al. 2021) (https://zenodo.org/records/10652104), while the rest were sham-operated. Rats were housed in groups of 3 per cage with ad libitum access to food (SAFE^®^ 150 laboratory animal diet) and water. In line with the 3R principles to promote animal welfare, each cage was enhanced with a cardboard tunnel for shelter, as well as gnawing and nesting materials. The rats were housed on a 12 h light/dark cycle with lights on from 7 a.m. to 7 p.m. Room temperature was maintained at 23 °C. All experimental interventions were conducted during the light cycle.

The Supplementary Material & Methods contains a detailed description of the Materials and Methods section together with information about the number of animals used for each experiment (Table S1-S3).

### Biochemical measurements to validate the CLD

Liver parameters (plasma bilirubin, aspartate aminotransferase (AST/GOT) and alanine aminotransferase (ALT/GPT)), glucose (Glc) and blood NH_4_^+^ were measured longitudinally until week-8 post-BDL (Table S1 and Fig. S3).

### In-vivo metabolic changes using ^1^H-MRS

^1^H-MRS spectra were acquired on the 9.4 T system, as previously described (Braissant et al. 2019), before (week-0) and at weeks-2, 4, 6, 8 post-BDL, each animal being its own control. Three different volumes of interest were selected in hippocampus (2 × 2.8 × 2mm^3^), cerebellum (2.5 × 2.5 × 2.5mm^3^), and striatum (2.5 × 2 × 2.5mm^3^), with 8 to 23 rats/week scanned. LCModel was used for quantifying 18 metabolites. The number of animals per time point varied due to loss of some animals between 6 and 8 weeks because of disease progression, use of multiple longitudinal cohorts, planned euthanasia for histological analysis and inclusion of measurements for striatum only for later cohorts (Table S2).

### Cellular morphological changes using histology

#### Immunohistochemistry (IHC)

Animals were sacrificed for histological evaluation at week-4 and 8 (*n* = 3 BDL per time point, *n* = 3 sham). Brains were removed and fixed in 4% formaldehyde PBS solution overnight at 4 °C, followed by PBS wash and 48 h cryopreservation in 30% sucrose in PBS at 4 °C. They were embedded in an optimum cutting temperature compound (4583, Tissue-Tek^®^ O.C.T. Compound, SAKURA) and then flash-frozen using dry ice and 2-Methylbutane (M32631, Sigma-Aldrich) for sectioning. Sections were mounted on Superfrost Plus microscope slides (22037246, Thermo Scientific). After the staining procedure, the slides were mounted with ProLong™ Diamond Antifade Mountant (P36970, Thermo Fisher) and covered with coverslips.

IHC analysis was carried out using immunofluorescence (IF) detection. Mouse monoclonal anti-GFAP antibody (MAB360 Merck Millipore, RRID: AB_11212597) (2 h at RT) at 1/100 dilution with secondary Alexa Fluor^®^ 594-AffiniPure Rat Anti-Mouse IgG (H + L) antibody (415–585−166 Jackson ImmunoResearch Europe Ltd.,) RRID: AB_2340284 (1 h at RT) at 1/200 dilution were used to characterize the astrocytes morphology(Louth et al. 2017). Iba1 goat polyclonal antibody (PA5-18039 Thermo Fisher, RRID: AB_10982846) (2 h at RT) at 1/100 dilution with Alexa Fluor™ Plus 488 Donkey anti-Goat (A32814, Thermo Fisher, RRID: AB_2762838) secondary antibody (1 h at RT) at 1/500 dilution were used to characterize the microglia morphology. Nuclei were stained with DAPI (D1306, Thermo Fisher).

Morphometric Sholl analysis was performed as previously described (Braissant et al. 2019). Protoplasmic astrocytes and microglia of each group of BDL and sham rats were randomly traced for all GFAP and Iba1-positive processes, respectively, in the hippocampus, cerebellum, and striatum (7 sections/hemisphere). Detailed information regarding the number of analyzed cells and processes is provided in the figure captions.

Golgi Cox staining was applied at 8-weeks post-BDL/sham (*n* = 6 BDL, *n* = 6 sham) to analyze the neuronal cytoarchitecture as previously described (Giovannoni et al. 2024). Brains were cut into 115 μm-thick sagittal sections using Leica VT1200 S vibratome. Tissues with homogeneous staining and clearly visible dendritic segments and spines were chosen for quantitative analysis (25 sections/hemisphere). The surface of the neuronal soma was measured, and manual counting of the dendrite spine was conducted(Giovannoni et al. 2024). Measurements were obtained from the dentate gyrus (DG) granule cells (BDL soma ~ 200 cells, apical dendrites ~ 150; Sham soma ~ 180 cells, apical dendrites ~ 80) and CA1 neurons (BDL soma ~ 200 cells, apical and basal dendrites ~ 100 each; Sham soma ~ 120 cells, apical and basal dendrites ~ 60 each) of the hippocampus and Purkinje (Pj) cells (BDL soma ~ 500 cells, apical dendrites ~ 220; Sham soma ~ 180 cells, apical dendrites ~ 80) in the cerebellum. Golgi-Cox staining was compromised in the basal ganglia region, precluding reliable assessment of the striatum.

Sections (IHC and Golgi Cox) were digitized using a MEIJI TECHNO TC5600 microscope with an INFINITYX-32 camera. To provide comprehensive analysis, images were captured using 5x, 10x, 20x, and 50x objectives. Images were acquired and analyzed using the INFINITY ANALYZE 7 software (Lumenera, Canada).

#### Statistical analysis

The results of ^1^H-MRS (metabolite concentrations) are presented in three different ways: bar plots, scatter plots and 1 st axis of STATIS analysis. All results in the bar plots are presented as mean ± SD.

Given the complexity and number of the variables, we opted for a STATIS multi-table principal component analysis to evaluate the contribution of each metabolite to the axis of maximum variance of the data, here mainly representing the time evolution during the disease (1st Axis). The pipeline for application of the STATIS method was implemented as previously described^10,11^ using RStudio^9^ (for details see Supplementary Materials and Methods).

For metabolites and the blood values, one-way ANOVA (Prism 5.03, Graphpad, La Jolla CA USA) followed by Bonferroni’s multiple comparisons post-tests were used. Differences between brain regions were assessed using two-way ANOVA. The % change was calculated in comparison to week-0. Further details are provided in Supplementary Materials and Methods.

For histology, a one-way ANOVA followed by post-hoc Turkey HSD was used to compare BDL and sham-operated rats. All tests were 2-tailed.

## Results

### BDL-induced differential brain regional changes in osmolytes, neurotransmitters and antioxidants measured by in-vivo ^1^H-MRS

**Fig. 1:**
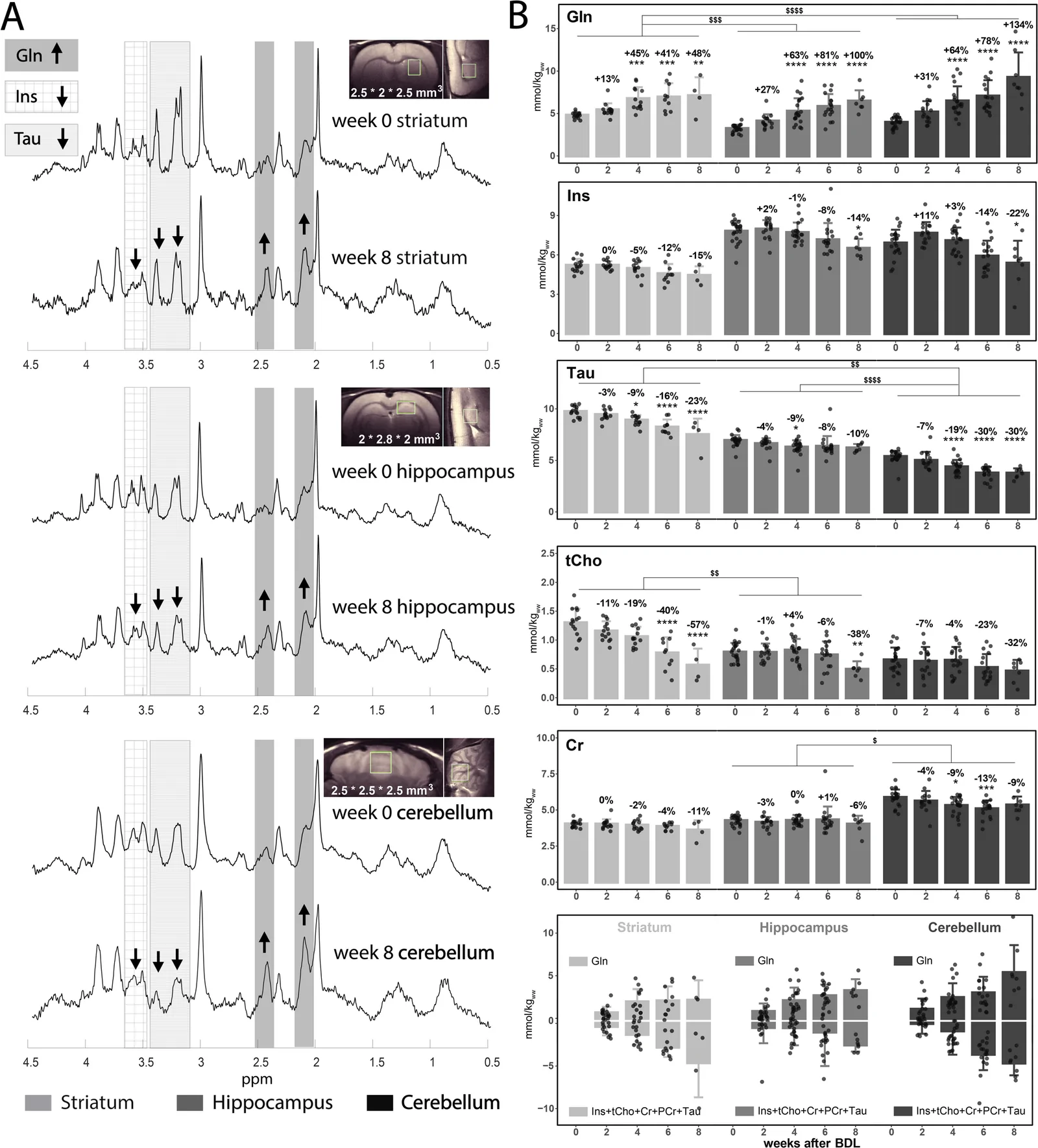
(**A**) Representative in-vivo ^1^H-MRS spectra from 1 animal at week-0 (before BDL) and at post-operative week-8 in striatum, hippocampus, and cerebellum. Increased Glutamine (Gln) and decreased Inositol (Ins) and taurine (Tau) can be observed. (**B**) Longitudinal ^1^H-MRS evolution of central nervous system (CNS) osmolytes. (Data points: metabolite concentrations for each measured animal after quality control) Last panel: Absolute Gln increase compared with the decreased sum of other main osmolytes. Mean$$\:\pm\:$$SD; *p < 0.05, **p < 0.01, ***p < 0.001, ****p < 0.0001 (1-way ANOVA). Brain regions differences: ^$^*p* < 0.05, ^$$^*p* < 0.01, ^$$$^*p* < 0.001, *p* < 0.0001 (two-way ANOVA))

Chronologically, the first common neurometabolic finding was an increase in Gln concentration at week-2 post-BDL. This was observed in the three brain regions, reaching significance at week-4 post-BDL (striatum + 41%, hippocampus + 63%, cerebellum + 64%) with a rise in concentration observed until week-8 post-BDL. Gln increase was strongest in the cerebellum (+ 134% at week-8; Fig. 1A & B, Fig. S4). Brain Gln correlated strongly for all brain regions with blood NH_4_^+^ (*p* ≤ 0.0001, Fig. S5).

As Gln increased the other main CNS osmolytes - myo-inositol (Ins), taurine (Tau), Cr and tCho - decreased (Fig. 1B, Fig. S4). Specifically, Ins decreased consistently over time in the three regions reaching significance at week-8 post-BDL for the cerebellum (−22%), hippocampus, and striatum (−14% and − 15%). tCho also decreased consistently in the three brain regions, with the striatum showing the strongest reduction (−40%) at week-6 post-BDL, continuing to decrease until week-8 post-BDL. Tau decreased consistently over time reaching significance at week-4 post-BDL for all brain regions (striatum − 9%, hippocampus − 8%, cerebellum − 19%), and continued to decrease till week-8 post-BDL. This decrease was statistically strongest in the cerebellum when compared to the hippocampus and striatum. Cr also decreased consistently in all studied brain regions, most notably in the cerebellum (−9% at week-8 post-BDL). Ins and tCho correlated significantly with both Gln and blood NH_4_^+^ (in all brain regions, Fig. S5). Brain Gln showed a significant correlation with Tau for the hippocampus and cerebellum while blood NH_4_^+^ correlated significantly with Tau in all brain regions. Similarly, Gln correlated significantly with Cr in the hippocampus and cerebellum, while the correlation with blood NH_4_^+^ was not confirmed here.

Despite the smallest absolute increase in Gln, the striatum showed a more pronounced decrease of the absolute concentration of the main osmolytes (Ins+tCho + Cr+PCr + Tau) (8-weeks post-BDL: Gln + 2.3 mmol/kg_ww_ vs. sum of osmolytes − 4.2 mmol/kg_ww_) than the hippocampus (8-weeks post-BDL: Gln + 3.2 mmol/kg_ww_ vs. sum of osmolytes − 2.6 mmol/kg_ww_) and the cerebellum (8-weeks post-BDL: Gln + 5.3 mmol/kg_ww_ vs. sum of osmolytes − 4.1 mmol/kg_ww_) (Fig. 1B).

**Fig. 2:**
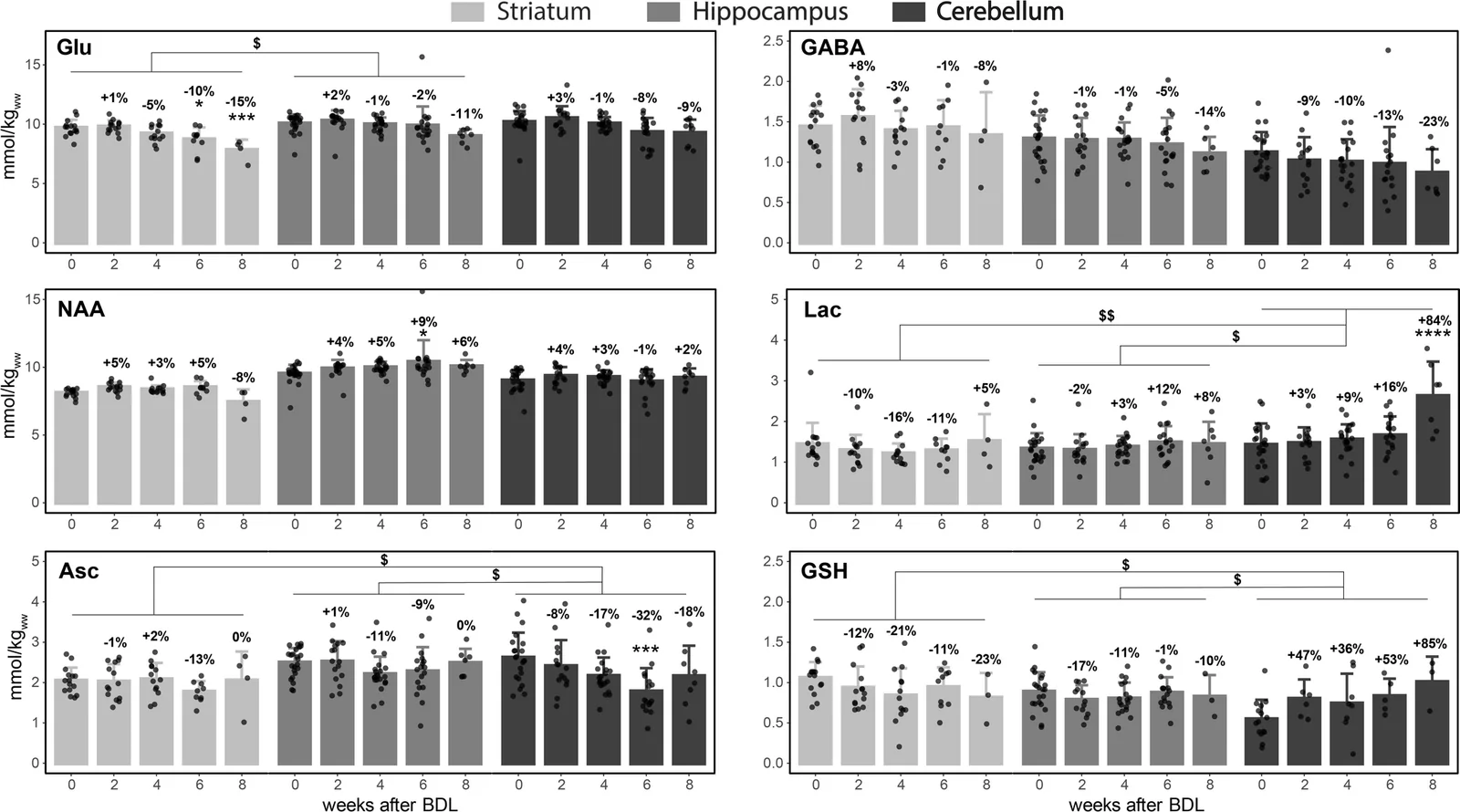
Longitudinal ^1^H-MRS evolution of other brain metabolites (Data points: metabolite concentrations for each measured animal after quality control). Mean$$\:\pm\:$$SD; *p < 0.05, **p < 0.01, ***p < 0.001, ****p < 0.0001 (1-way ANOVA). Brain regions differences: ^$^*p* < 0.05, ^$$^*p* < 0.01, ^$$$^*p* < 0.001, *p* < 0.0001 (two-way ANOVA))

The neurotransmitter Glutamate (Glu) showed a decrease in the later stages of the disease, reaching a significant − 10% at week-6 post-BDL in the striatum and continued to decrease until week-8 post-BDL in the three brain regions (Fig. 2, Fig. S6). Glu correlated stronger with blood NH_4_^+^ (significant for the three brain regions), than with Gln where the correlation was weaker showing significance for striatum and cerebellum only (Fig. S7). Aspartate (Asp, data not shown) and $\:\gamma\:$**-**aminobutyric acid (GABA) displayed no significant changes. However, GABA displayed an overall tendency of decrease especially in the hippocampus and cerebellum and correlated significantly only with cerebellar Gln.

No significant changes in neuronal marker NAA were observed, however, a tendency to decrease in the striatum was noted. Lactate (Lac) showed a striking increase of 84% only in the cerebellum in the final stage of the disease (week-8 post-BDL) (Fig. 2).

The antioxidant ascorbate (Asc) decreased significantly in the cerebellum at week-6 post-BDL (−32%). Glutathione (GSH) displayed no significant changes throughout the disease, with a tendency of increase in the cerebellum only (Fig. 2, Fig. S6).

### Brain Gln and blood NH_4_^+^ contribute to the variance in STATIS analysis of ^1^H-MRS and blood data

**Fig. 3:**
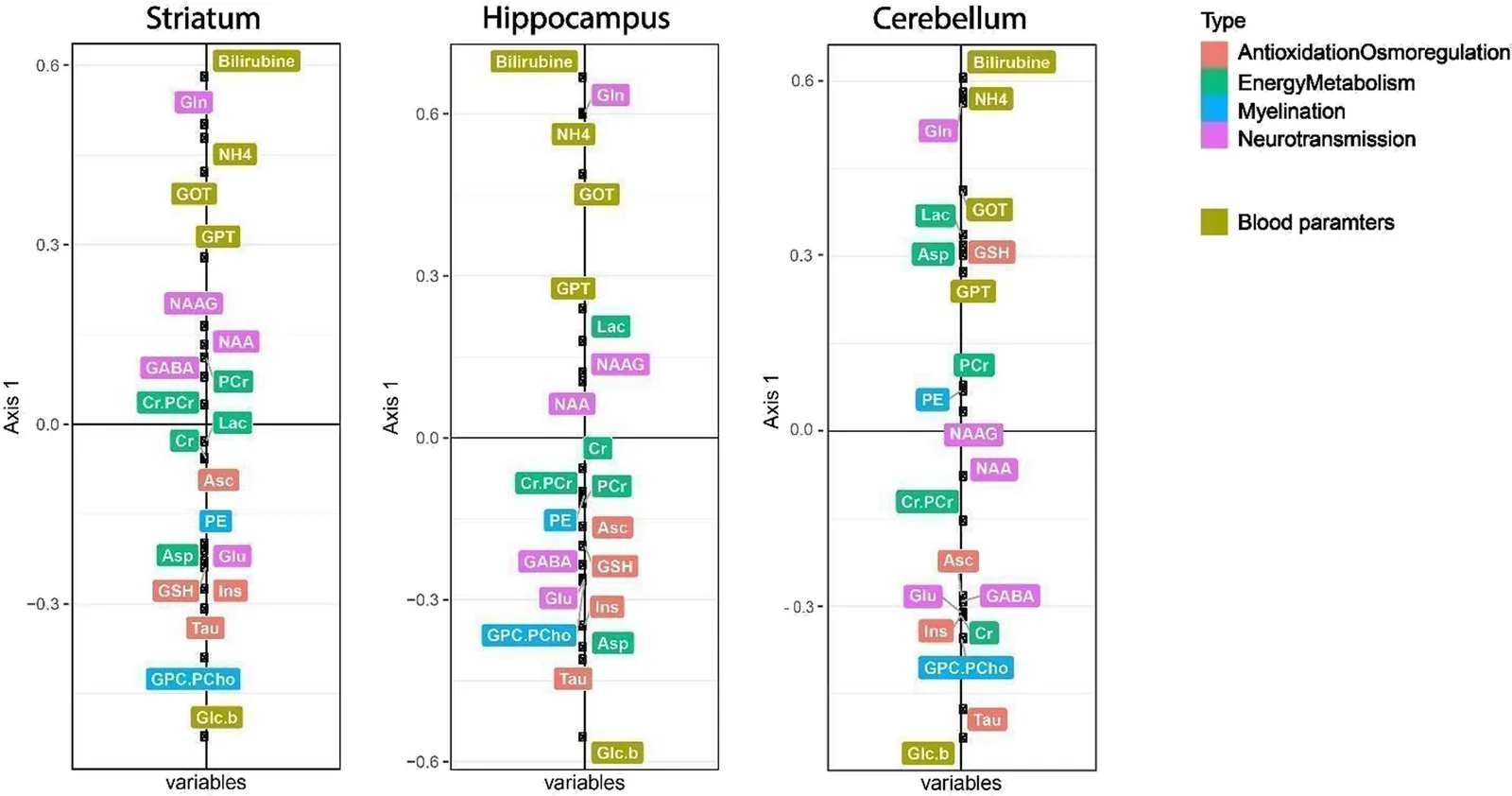
The first axis resulting from the STATIS analysis. The axis corresponding to the dataset containing brain metabolites + blood parameters. Metabolites that highly contribute to the 1 st Axis (towards the extremities) are the ones driving the variance and they can be grouped based on their contribution. The variables that are positioned closer to the extremes of the axis are the ones highly contributing to the variance explained by the axis (variance of the longitudinal evolution of metabolites during the disease in a specific brain region), while the variables positioned at the opposite extremities showed opposite changes

The longitudinal measurements of metabolic profiles and blood biochemistry of all animals were analyzed altogether using STATIS (separated by brain region). The 1 st Axis of principal component analysis represents the variance of the dataset brought on by the time evolution of the metabolites during disease. The metabolites with a high loading (positioned towards the extreme of the axis) are important, highly contributing to the 1 st Axis and driving the variance. Increase of bilirubin and ammonia as well as decrease of blood Glc are known, longitudinally observed changes during CLD and confirmed here (Fig. S3) with their high contributions to the 1 st Axis (Fig. 3). Therefore, we can take them as a reference by putting the contributions of brain metabolites into the context of known changes during CLD. Gln has a high contribution to the variance (high contribution to the 1 st Axis) for all brain regions and behaves similarly to bilirubin and NH_4_^+^ (Fig. 3). On the opposite side of Gln we have Ins, Tau and tCho (GPC+PCho), which showed a decrease consistent with osmolyte regulation. The strong contribution of tCho to the 1 st Axis in the striatum corresponds to its strongest decrease as shown in Fig. 1B. For the cerebellum, a contribution of Lac is observed (on the same side as Gln) corresponding to a strong increase of Lac at week-8 in this brain region.

### BDL-induced brain regional changes in astrocytes, microglia and neurons

**Fig. 4:**
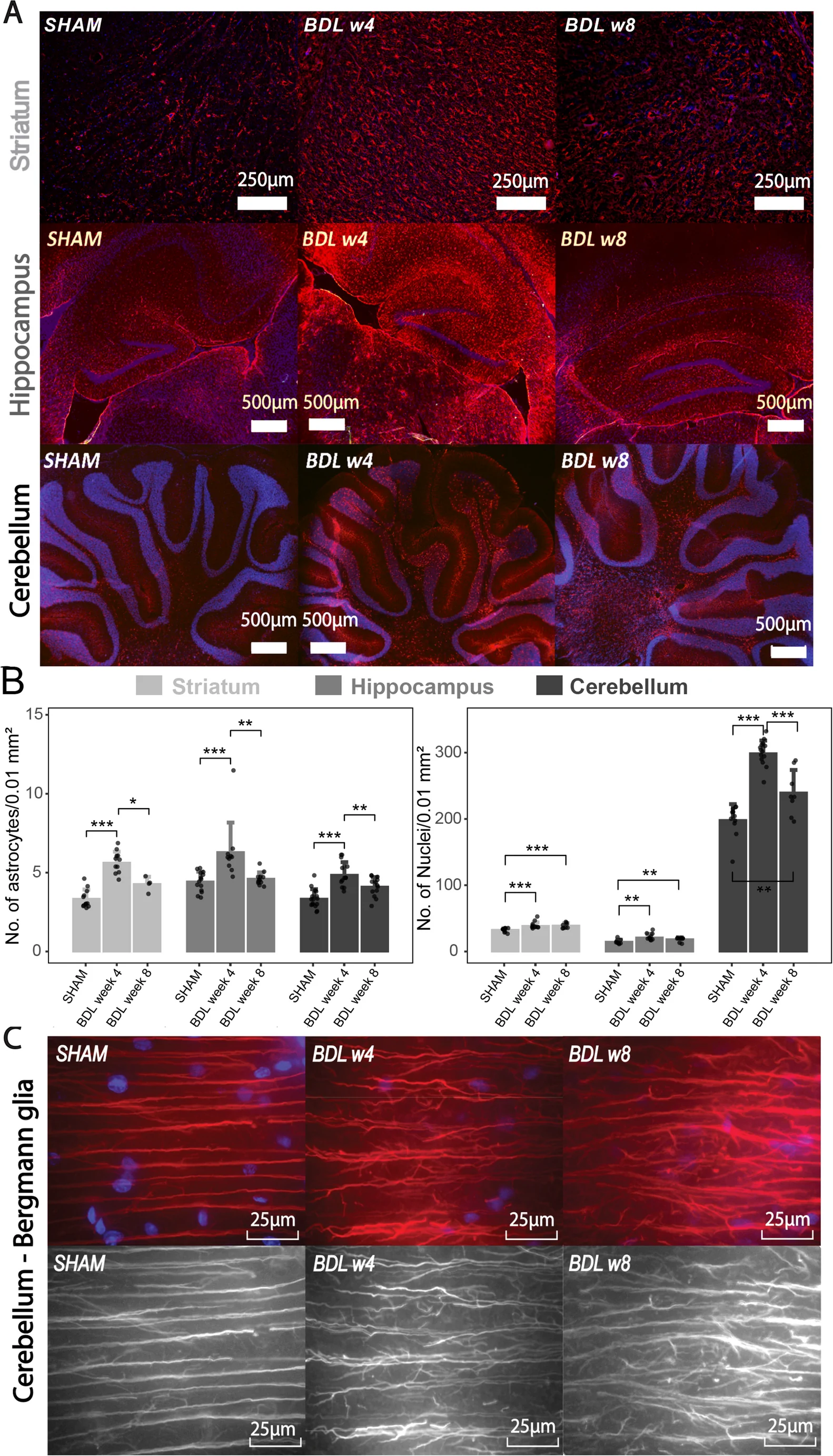
Anti-GFAP (red) and DAPI (blue) staining of hippocampus and cerebellum: (**A**) Representative micrographs of sham, week-4 and 8 post-BDL, and (**B**) the astrocytes density quantification (integer cell and nuclei counts per ROI were normalized to a standard area of 0.01 mm², and ROI was placed only on uniformly stained tissue). Note the significant increase in astrocytes number and total (all DAPI-stained) cell nuclei number at week-4 post-BDL. (**C**) Cerebellum - Bergmann glia: fibers deterioration and expansion of end-feet through the pial surface starting at BDL week-4 that increased in severity at week-8. Seven sections per rat (3 rats per group; ~250 μm spacing) were analyzed. One-way Anova with post-hoc Tukey HSD, *p < 0.05, **p < 0.01, ***p < 0.001, ****p < 0.0001, mean ± SD

**Fig. 5:**
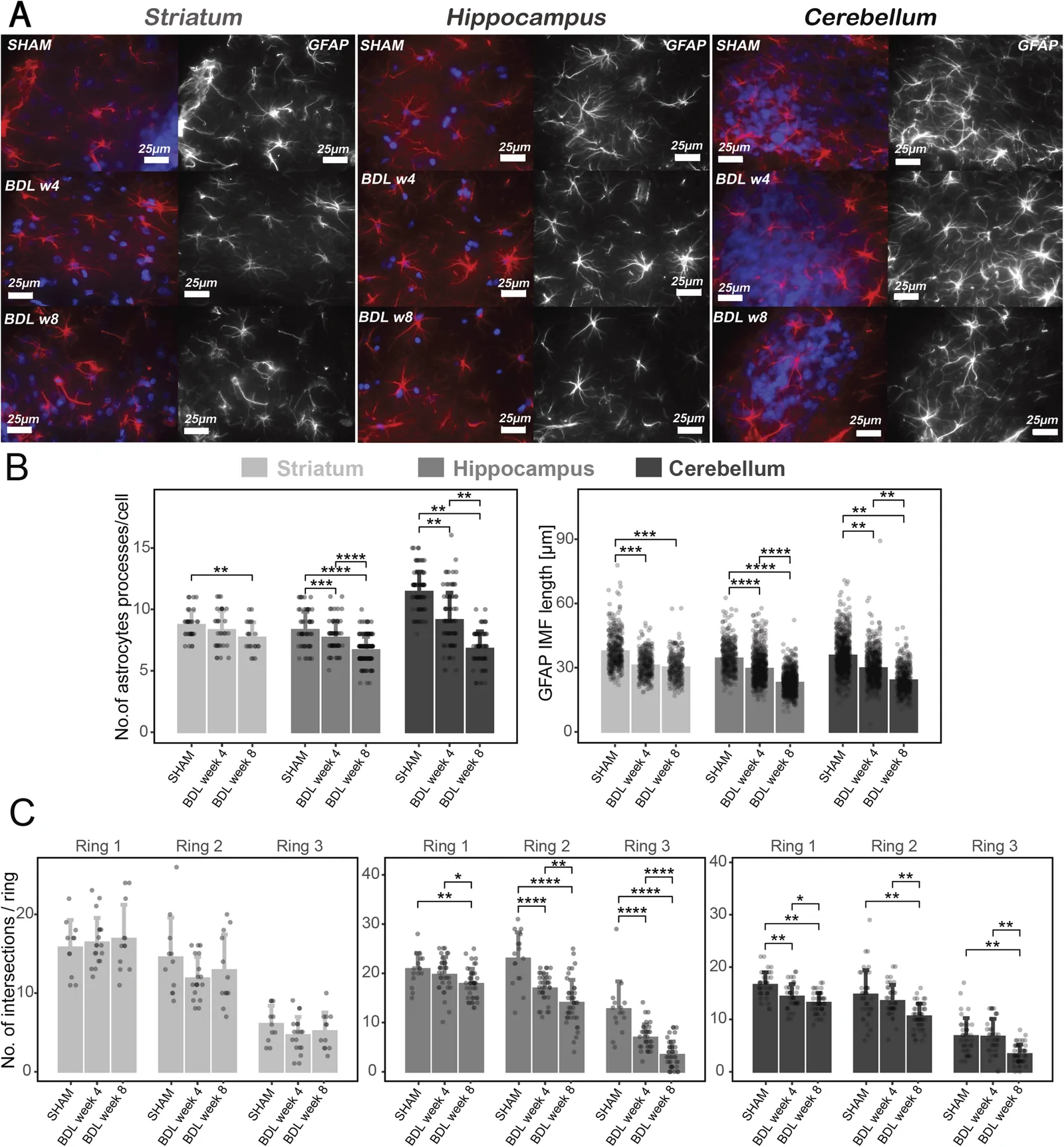
Anti-GFAP (red) and DAPI (blue) staining. Astrocytic morphology analysis: (**A**) Brain sections from sham and BDL rats at 4 and 8-weeks post-BDL (hippocampus, cerebellum, and striatum). (**B**) Astrocytic processes number (left panel) and process lengths (right panel) quantification, and **(C)** the number of intersections across the given rings starting from the cell body’s center - plotted according to subregions (1–3) (Sholl analysis, Fig. S1). Data points explanation: The number of data points varies due to differences in analyzable ROIs (cutting plane and staining quality); only uniformly stained, well-separated cells were included for Sholl ring analysis. Seven sections per rat (3 rats per group; ~250 μm spacing) were analyzed. *Striatum*: ~30 astrocytes/group for process number (~ 350 processes/group); ~14 astrocytes/group for Sholl (ring) analysis. *Hippocampus*: ~120 astrocytes/group for process number (~ 750 processes/group); ~30 astrocytes/group for Sholl (ring) analysis. Cerebellum: ~90 astrocytes/group for process number (~ 700 processes/group); ~40 astrocytes/group for Sholl (ring) analysis. One-way Anova with post-hoc Tukey HSD, *p < 0.05, **p < 0.01, ***p < 0.001, ****p < 0.0001, mean ± SD

A significant increase in astrocytes (GFAP+ cells, astrocytosis-reactive astrocytic response) and total (all) cell nuclei number was observed at 4-weeks post-BDL in the hippocampus (+ 48%, 45%), cerebellum (+ 45%, 53%), and striatum (+ 31%, 21%), respectively (Fig. 4B). Although a significant reduction of astrocytosis was observed at week-8 when compared to week-4 post-BDL (hippocampus: +48% to + 8%; cerebellum: +45% to + 23%, and striatum: +31% to + 15%, Fig. 4B), the number of astrocytes remained slightly increased compared to sham. Furthermore, vesicular nuclei with migrated chromatin, characteristic of Alzheimer type II astrocytes, were observed in all brain regions at 4-weeks post-BDL surgery (Fig. S8). Sholl analysis depicted a time-dependent reduction of the number of processes at week-4 (hippocampus − 6%, cerebellum − 20%, and striatum − 5%) and continued to decrease until week-8 post-BDL (Fig. 5B) with the cerebellum showing a stronger reduction in the number of processes. In the striatum the decrease became significant only at week-8 (−12%). The length of astrocytic processes decreased over time, reaching significance at week-4 for all brain regions (hippocampus − 13%, cerebellum − 17%, striatum − 18%, Fig. 5B). A significant decrease in the astrocytic processes intersections in the hippocampus and cerebellum was observed at 4-weeks, displaying a stronger decrease at week-8 (hippocampus: −14%-Ring1, −39%-Ring2, −72%-Ring3, cerebellum: −21%-Ring1, −28%-Ring2, −51%-Ring3, Fig. 5C). No significant decrease of intersections number was observed for the striatum (Table S4). Processes of Bergmann glia (BG), specialized astrocytes found in the cerebellum, also exhibited morphological alterations displayed by a significant fiber deterioration at 4-weeks post BDL (Fig. 4C).

**Fig. 6:**
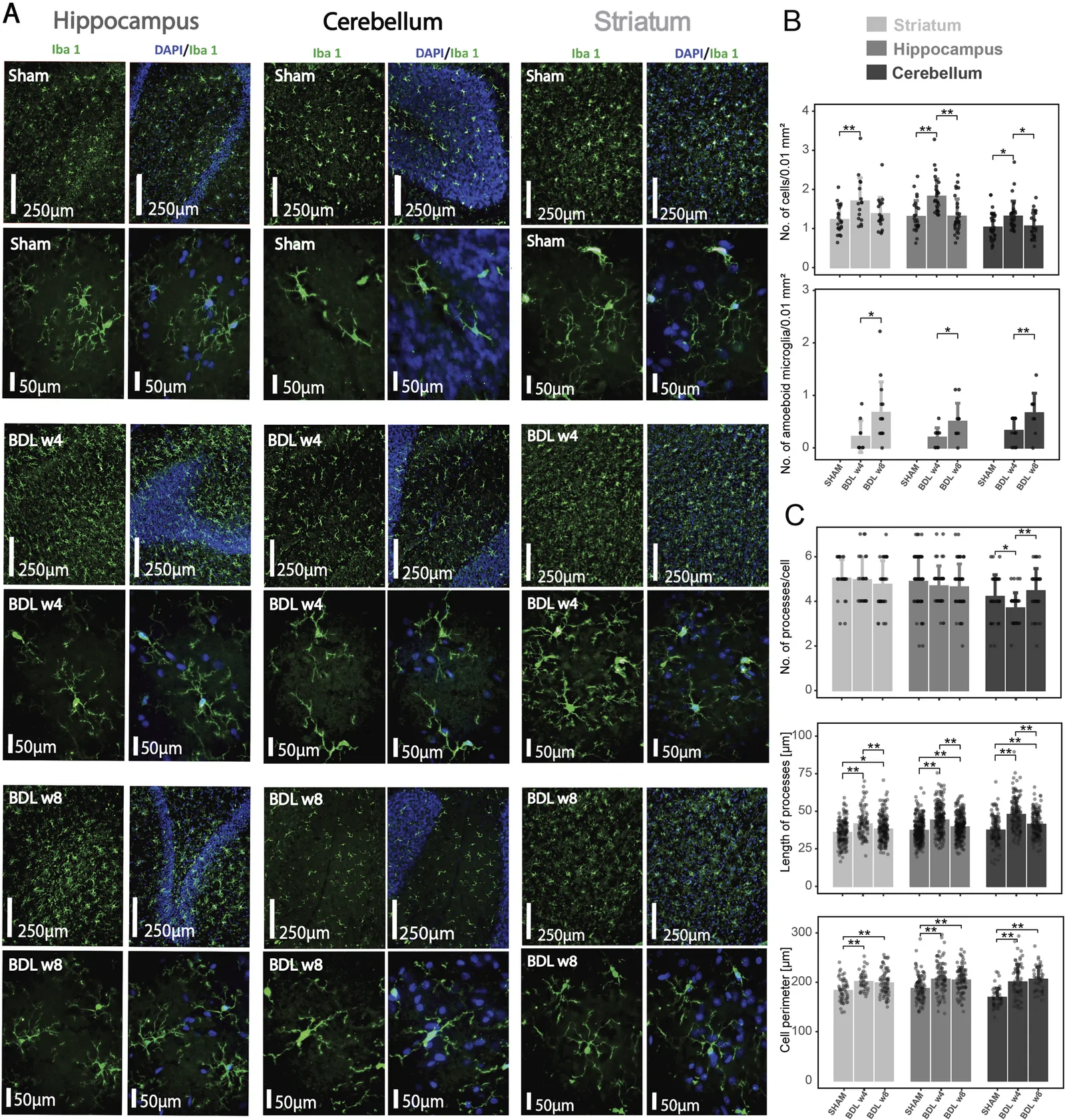
Anti-Iba1 (green) and DAPI (blue) staining of microglia: (**A**) Brain sections from sham and BDL rats at 4 and 8-weeks post-BDL (hippocampus, cerebellum, and striatum). (**B**) Microglia density and amoeboid microglia quantification (integer cell counts per ROI normalized to a standard area of 0.01 mm², and ROI was placed only on uniformly stained tissue). (**C**) Microglia processes number, processes length, and cell perimeter analysis. Data points explanation: The number of data points varies due to differences in analyzable ROIs (cutting plane and staining quality); only uniformly stained, well-separated cells were included for Sholl perimeter analysis. *Striatum*: ~30 microglia/group for process number (~ 160 processes/group); ~50 microglia/group for Sholl (perimeter) analysis. *Hippocampus*: ~60 microglia/group for process number (~ 240 processes/group); ~90 microglia/group for Sholl (perimeter) analysis. Cerebellum: ~40 microglia/group for process number (~ 130 processes/group); ~50 microglia/group for Sholl (perimeter) analysis. One-way Anova with post-hoc Tukey HSD, *p < 0.05, **p < 0.01, ***p < 0.001, ****p < 0.0001, mean ± SD

Microglia morphological changes and macrophage activation were depicted using Iba1 staining. A significant increase in Iba1 + cells was detected at 4-weeks post-BDL (hippocampus + 39.9%, cerebellum + 27.2%, striatum + 38.6%, (Fig. 6A & B, Fig. S9). At week-4 post-BDL, microglia were morphologically altered, with increased process length and cell perimeter (hippocampus: +18.6%, + 10%, cerebellum: +27%, + 18.5%, striatum: +16%, + 9.8%, processes length and cell perimeter, respectively) (Fig. 6C). At week-8 post-BDL a significant reduction of microglia Iba1 + cells was observed in all brain regions and was accompanied by a significant increase of amoeboid microglia number (Table S4).

**Fig. 7:**
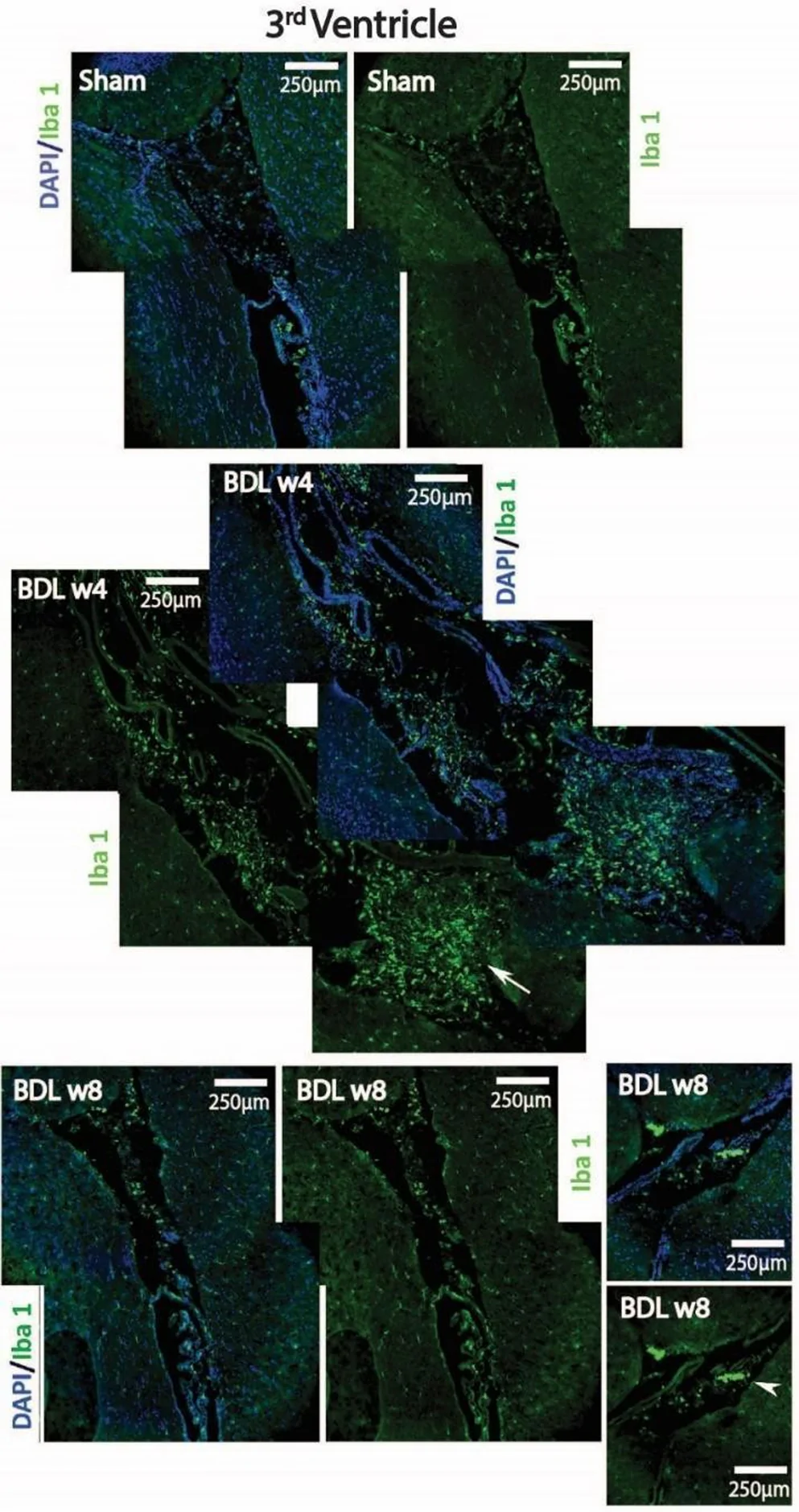
2D reconstruction of anti-Iba1 (green) and DAPI (blue) staining of diencephalon (3rd ventricle): Activated macrophages accumulation and clot-like formation. Arrow - increased number of macrophages; arrowhead - macrophage clot-like formation. Stitched reconstruction was generated only for the BDL samples, where an increased presence of macrophages was observed and therefore needed a broader field of view

In sham, sparse Iba1 + phagocytically active macrophages were located in the choroidal stroma, in the 3rd and lateral ventricles, and in the hindbrain (aqueduct) choroid plexus. Starting 4-weeks post-BDL, a significant increase in Iba1+/activated macrophages was identified in the stroma as well as in the base of choroidal epithelial cells, with an increase in activated macrophages in the lateral ventricles at week-8 post-BDL compared to week-4. At week-8 post-BDL clot-like macrophage formations were present in the 3rd and lateral ventricles and hindbrain choroid plexus (Fig. 7, Fig. S9).

**Fig. 8:**
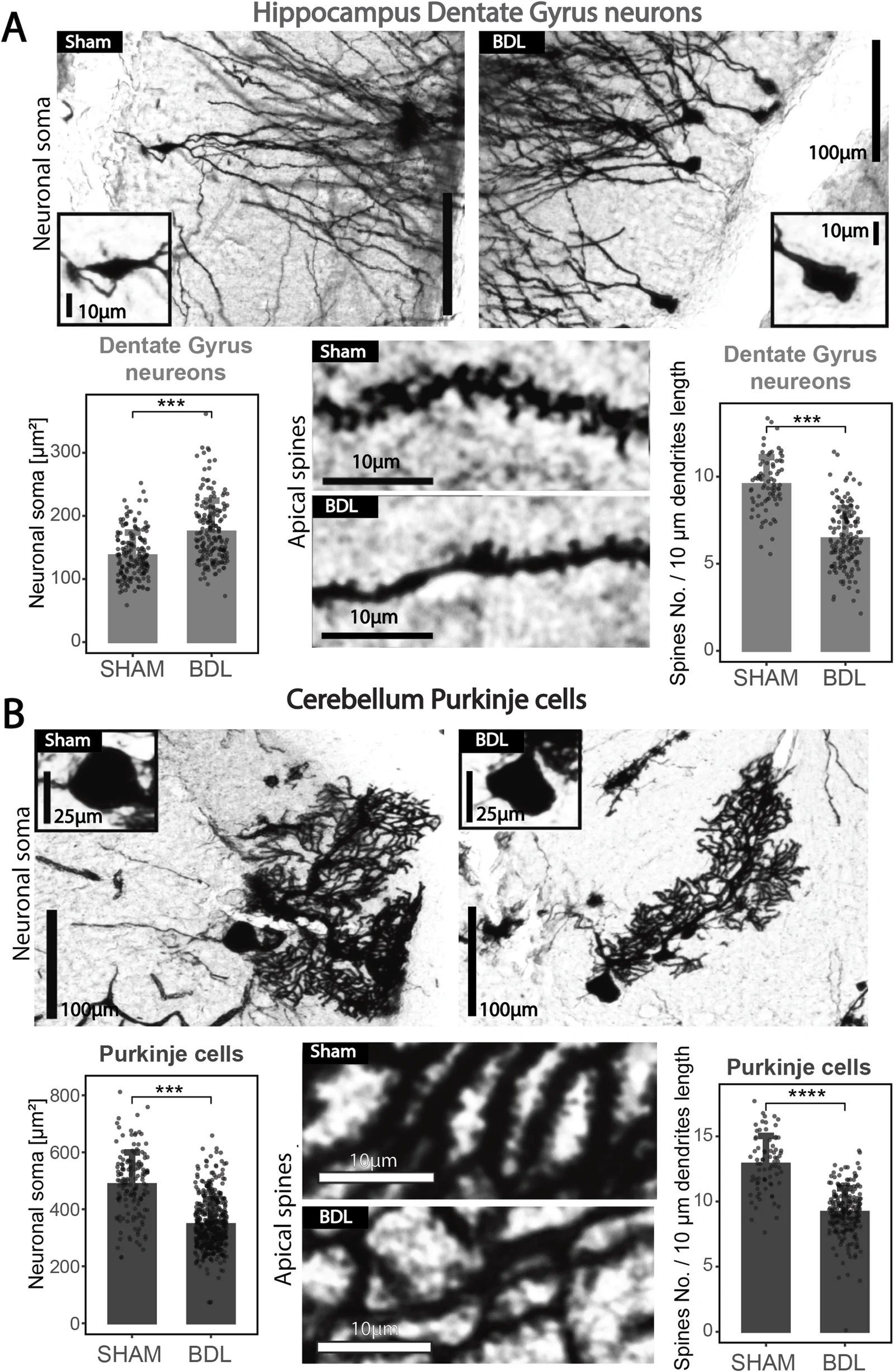
Golgi-Cox staining: Representative micrographs of Golgi-Cox staining: neuronal morphology analysis. In BDL the hippocampal Dentate Gyrus neurons (Data points: BDL soma ~ 200 cells, apical dendrites ~ 150; Sham soma ~ 180 cells, apical dendrites ~ 80) (**A**) and cerebellum Purkinje cells (Data points: BDL soma ~ 500 cells, apical dendrites ~ 220; Sham soma ~ 180 cells, apical dendrites ~ 80) (**B**) showed altered soma size and decreased dendritic spine density. One-way Anova with post-hoc Tukey HSD): *p < 0.05, **p < 0.01, ***p < 0.001, ****p < 0.0001

Changes in neuronal structure were revealed using Golgi-Cox staining: a significant increase in CA1 (+ 67%) and DG (+ 54%) neuronal soma surface and a significant loss of dendritic spines density in CA1 (−49%, apical and basal) and DG (apical − 43%) hippocampal neurons (Fig. 8A, Fig. S10, Table S5). In contrast to hippocampal neurons, Purkinje cells in the cerebellum showed a significant decrease of the neuronal soma surface (−22%) and dendritic spine density depletion (apical − 24% Fig. 8B).

## Discussion

In this unique study combining longitudinal in-vivo ^1^H-MRS in a recognized rat model of CLD-induced type C HE and ex-vivo analysis of brain cell subtypes, we demonstrated that there are shared and also differential metabolic and cellular responses to CLD in the cerebellum, hippocampus, and striatum. We showed that metabolically Gln displayed the most rapid and stronger change in all the brain regions, thus was pointed as a surrogate of the disease. This was followed by a decrease in osmolytes, neurotransmitters and changes in antioxidants across regions. Moreover, cerebellum exhibited the heaviest Gln burden, along with distinct changes such as Lac and GSH increase, and Asc and GABA decrease, while the striatum displayed the lowest Gln increase but the strongest reduction in osmolytes (e.g. tCho). At the cellular level, beyond the well-known astrocytic alterations characteristic of HE, several regional differences were observed. The cerebellum displayed a stronger reduction in astrocytic processes. Furthermore, microglia displayed morphological alterations, including changes in processes length and perimeter, and neurons exhibited region-specific soma size alterations (reduced in Pj cells (cerebellum), increased in DG and CA1 (hippocampus)), and decreased spine density (slightly stronger in the hippocampus). The striatum seemed to display weaker changes in astrocytes morphology potentially reflecting lower Gln increase but stronger decrease in osmolytes, warranting further investigations.

Although the contribution of these alterations to neurodegeneration and functional deficits in HE still needs to be clarified, our findings highlight the value of ^1^H-MRS as a non-invasive, i*n-vivo* approach for elucidating the region-specific brain alterations in HE. While this work was performed in the rodent brain, the same ^1^H-MRS approach can be used in humans. Its application in both clinical and preclinical settings provides critical insights into the pathophysiological heterogeneity of HE, supporting its role as a valuable tool for linking neurochemical changes to the clinical spectrum observed in patients.

### Common changes among the three studied regions

Longitudinal measurements of CNS metabolic profiles and blood biochemistry identified the brain regional and blood ‘drivers’ of the disease. Blood measurements showed a systemic rise of bilirubin and NH_4_^+^, consistent with previous observations in CLD (Häussinger et al. 2000; Braissant et al. 2019). Parallel longitudinal ^1^H-MRS measurements in the striatum, hippocampus, and cerebellum uncovered common neurometabolic alterations, with Gln as the first globally increased metabolite in all three brain regions, while stronger in the cerebellum. STATIS analysis revealed that Gln, NH_4_^+^ and bilirubin, similarly drive the variance of the dataset (Fig. 3) confirming that elevated blood NH_4_^+^ induces a rapid cascade of CNS metabolic events initiated by Gln increase. Moreover, recent studies on treated BDL rats (rifaximin and the probiotic VIVOMIXX (Rackayová et al. 2021; Flatt et al. 2021) identified Gln as the fastest-responding metabolite to treatment (lower increase), even without NH_4_^+^ changes. This highlights the importance of Gln in type C HE and the role of in-vivo ^1^H-MRS for therapy monitoring, in agreement with a recent meta-analysis study highlighting ^1^H-MRS in the assessment of HE in human patients (Zeng et al. 2020). Furthermore, a previous study reported no significant differences in brain water content or plasma ammonia levels between BDL and sham-operated rats (Jover et al. 2006), highlighting the need for other markers, especially at early stages. Consistently, we found no neurometabolic changes in children with compensated liver disease (^1^H-MRS at 7 T) (Cudalbu et al. 2023) but marked Gln elevation in a child with a congenital portosystemic shunt (Chabbey et al. 2024).

The brain is very susceptible to excess ammonia and it primarily depends on its clearance by Gln synthesis (via the glutamine synthetase enzyme (GS) in astrocytes to prevent secondary neurological damage (Brusilow et al. 2010). In disease conditions, the observed elevated Gln is largely situated in astrocytes causing an osmotic imbalance and a gradual decrease of other brain osmolytes (Häussinger et al. 2000; Córdoba et al. 2002; Braissant et al. 2019) (i.e. Ins, tCho, Tau and Cr Fig. 1B). Taken together, this neurometabolic pattern was observed in all of the investigated brain regions and can be considered another disease-wide feature.

An increase in astrocyte number at 4-weeks post-BDL was among the first global cellular changes observed across all studied regions. This increase is a hallmark of astrocyte reactivity and could be related to the stimulation of mature astrocytes and reentering into the proliferation cycle, a common feature in neuropathological disorders (Zhou et al. 2019). In parallel, the osmotic stress together with OS and other factors, likely contributed to the reported astrocytic morphological changes (processes length and number) (Simicic et al. 2022). Consistent with previous findings (Butterworth 2003), GFAP expression decreased at 8-weeks post-BDL. Morphological alterations in astrocytes may impair their functionality and contribute to subsequent astrocytic and then brain functional deficits (Braissant et al. 2019; Jaeger et al. 2019). Our findings are consistent with a recent study that indicated a reduction in GFAP immunoreactivity in the cerebellum of BDL rats compared to Sham animals (Arenas et al. 2024). In addition, the observed decrease in the numbers of GFAP- and Iba1-positive cells may be attributed not only to downregulation of expression of these markers but also to loss of cytoskeletal filaments, leading to impaired cellular integrity and degradation (Simicic et al. 2022; Braissant et al. 2025). While it was beyond the scope of the current study to evaluate the cell death, there was a concomitant decrease in DAPI-stained nuclei from week 4 to week 8 post-BDL.

Microglia morphological shifts are associated with activation and a rise in pro-inflammatory cytokines (i.e.,TNF, IL-1β, IL-6) and the release of reactive oxygen species (ROS) (Xu et al. 2021). This cellular alteration was observed in all investigated brain regions. Furthermore, we detected the presence and rise in amoeboid microglia numbers consistent with our prior observation of brain IL-6 accumulation (Pierzchala et al. 2022) and in line with studies of post-mortem brain tissue from cirrhotic patients with HE, where microglial activation was associated with hypertrophied cell bodies and elevated IL-6 levels (Dennis et al. 2014). The presence of amoeboid microglia has been associated with neurodegenerative disorders such as Alzheimer’s, Parkinson’s, and Huntington’s disease, all of which are characterized by chronic neuroinflammation (Au and Ma 2017). Their increased number may also be linked to an increase in their phagocytic activity (phagocytosis of cellular debris) (Au and Ma 2017), indirectly suggesting M1 (pro-inflammatory) activation (Guo et al. 2022). These findings are indirect evidence of neuroinflammation and potential brain degeneration in type C HE (Ochoa-Sanchez et al. 2021). Furthermore, an important increase of Iba1-positive macrophages was observed together with the appearance of macrophage aggregates (‘clot-like’ formations), a sign of macrophage activation within the choroid plexus and ventricular compartments in response to neuroinflammatory stimuli (Shechter et al. 2013); these cells secrete cytokines and ROS and can infiltrate into the CNS (Cui et al. 2021). The increased abundance of Iba1-positive macrophages may also reflect the recruitment of peripheral macrophages into the choroid plexus/ventricular compartment, in addition to activation/proliferation of resident choroid plexus macrophages, consistent with previous studies in other models of HE and in HE patients (Balzano et al. 2021; Leone et al. 2022, 2023). Moreover, changes in glucose and Gln metabolism alter macrophage signaling, resulting in the formation of M1 macrophages and pro-inflammatory cytokines (IL-6, IL-1, and TNF) (Newsholme et al. 2023). Therefore, choroid plexus may also play an important role in the modulation of neuroinflammation in type C HE.

The ^1^H-MRS measurements revealed an overall decrease of Glu, an alteration that can have multiple causes, including increased synthesis of Gln from NH_4_^+^ which could contribute to altered neurotransmission (Cabrera-Pastor et al. 2019), or an NH_4_^+^ induced OS which might inhibit Glu uptake in astrocytes (Norenberg et al. 2009). Glu is a direct precursor of GABA synthesis via the enzyme glutamate decarboxylase (Bak et al. 2006; Jewett and Sharma 2024). We observed a decline in GABA concentration, most pronounced in the cerebellum. A finding supported by herein observed Glu decrease, which would result in reduced GABA synthesis, and may relate to morphological changes observed in the Purkinje cells. However, other studies have reported increased GABA synthesis (Sørensen et al. 2022) or elevated GABA concentrations in BDL rats and HE patients (Zöllner et al. 2023). These contradictory findings, together with GABA crucial role in cognitive function (Schmidt-Wilcke et al. 2018), highlight the need for future investigations since disturbances in neurotransmitters pathways have been related to learning and memory abnormalities observed in BDL rats (Leke et al. 2013).

The majority of HE research to date has focused on astrocyte morphology and biology, with minimal attention to neurons (Chen et al. 2014; Angelova et al. 2022; Tamnanloo et al. 2023). At week-8 post-BDL, we reported pronounced morphological changes of the hippocampal and cerebellar neurons, indicating for the first time significant region-specific alterations in soma size of hippocampal CA1 and DG neurons and the cerebellar Purkinje cells. The observed reduction in spine density, particularly in the hippocampus, is extremely relevant to brain connectivity and long-term synaptic plasticity, both being critical for cognitive function, and likely contribute to the neurological decline seen in HE. The significant decline of spine density in the Purkinje cells may relate to the increase of OS (Pierzchala et al. 2022), as highlighted by the changes in antioxidants and Glu decrease, which plays an important role in the activity-dependent astrocyte-neuron interactions (Zhang et al. 2017). Only a few recent studies briefly explored neurons in the context of type C HE (Angelova et al. 2022; Tamnanloo et al. 2023), their findings suggested the presence of neurodegeneration and neuronal loss, supporting our findings. Given that neuronal morphological changes can be linked with memory decline and cognitive dysfunction (Calabrese et al. 2006), these results offer the development of a novel research direction in HE. Furthermore, the observed astrocytic morphological changes may leave the neuronal network unprotected, leading to altered neuronal integrity, as demonstrated in the present study, and potentially resulting in long-lasting functional deficits.

### Differential brain regional changes

#### Cerebellum

Although Gln increased globally in the brains of BDL rats, the cerebellum showed the strongest increase, suggesting that cerebellar astrocytes carried the heaviest Gln load. In parallel, stronger astrocytic (processes) and microglial changes combined with a decreased Purkinje cell soma size and spine density (Fig. 8B) were observed, suggesting a distinct local response to the Gln load. It could be argued that the high cerebral blood flow and ammonia extraction factor reported in cirrhotic patients (Ahl et al. 2004), together with the comparatively high percentage of neurons and astrocytes in the cerebellum (Herculano-Houzel 2014), make this brain region more susceptible to ammonia. In fact, the cerebellum accounts for 70–80% of the total number of rat brain neurons (Herculano-Houzel 2010). Thus, the resulting high metabolic and neurotransmitter recycling demands may increase its susceptibility to ammonia-induced osmotic and oxidative stress and metabolic dysfunction. Furthermore, regional differences in vulnerability to ammonia intoxication could reflect the natural heterogeneity of astrocytes and their ability to remove ammonia. Previous studies showed regional differences in GS activity and GFAP expression, with the cerebellum and hippocampus displaying higher GS activity compared with the striatum (Norenberg 1979; Patel et al. 1985). As GS plays a major role in ammonia detoxification via synthesis of Gln, the differences in GS expression might contribute to differential responses in hyperammonemic conditions (Suárez et al. 2002; Cooper 2012) and consequently higher Gln accumulation in the cerebellum. Furthermore, the cerebellum’s tendency for stronger neuroinflammation (Taylor-Robinson et al. 1994; Ahl et al. 2004; Sartorius et al. 2008; Pierzchala et al. 2022) makes it increasingly affected by inflammatory cytokines, among other deleterious molecules (Rodrigo et al. 2010; Pierzchala et al. 2022).

The reduction of Purkinje cell dendritic spines in the cerebellum, along with morphological alterations in Bergmann glia, a key modulators of Purkinje cell synaptic transmission may be associated with synaptic degeneration/loss, potentially contributing to motor dysfunction observed in HE, such as gait abnormalities and tremors (Louis et al. 2014; Ruff et al. 2024). Additionally, because the cerebellum is a brain region involved in motion and coordination, we previously showed that BDL rats travelled shorter distances than sham-operated animals, consistent with previous studies in the same animal model and patients with HE (Felipo et al. 2014; Braissant et al. 2019; Flatt et al. 2021).

Tau showed a significantly stronger decrease in the cerebellum than in the other brain regions and correlated with the Gln increase, supporting its role in astrocytic osmoregulation (Ramírez-Guerrero et al. 2022), which is more prevalent in this brain region due to the heavier load of brain Gln. Cr also showed a stronger decrease, probably assuming its osmoregulatory role (Hanna-El-Daher and Braissant 2016). The high exposure to NH_4_^+^ can cause an inhibition of Cr synthesis in the CNS, contributing to its decrease (Braissant et al. 2008) and potentially impacting energy metabolism.

The behavior of Lac was significantly different in the cerebellum, with a peak at week-8 (+ 84%), a unique change confirmed by its higher contribution to the 1 st axis of STATIS analysis (Fig. 3). The previously published results of Lac changes are contradictory: an increase in Lac playing a role in the pathogenesis of brain edema in BDL rats (Bosoi et al. 2014) or a decrease of cortical extracellular Lac pointing towards its intracellular accumulation (Hadjihambi et al. 2017) and a decrease in Lac cortical concentration (Hadjihambi et al. 2022). However, a brain region-specific Lac behavior should not be excluded, as measured in the current study. Increased Lac production in astrocytes that feed the surrounding neurons, as additional fuel to Glc, under glutamatergic synapse activation (astrocyte-neuron lactate shuttle hypothesis) (Pellerin and Magistretti 1994), has been suggested as a possible pathway for Lac increase in HE (Rose 2010). However, we observed reduced dendritic spine density in Purkinje cells (forming glutamatergic synapses with parallel fibers from granular neurons) and a decreased Glu under BDL, suggesting a lower activity of the glutamatergic synapses. Moreover, we showed a highly decreased blood Glc (Pierzchala et al. 2022; Mosso et al. 2022) significantly driving the data variance (Fig. 3), with a twofold decrease in glucose uptake measured in the same animal model in the cerebellum and hippocampus in-vivo (Mosso et al. 2022). None of our findings support the increased Lac production by astrocytes to support neurons, as this would require increased Glc uptake. Altogether, our data most probably suggest that the significant increase of Lac in the cerebellum reflects intense cellular stress, both neuronal and glial, at this late stage of disease, i.e. 8-weeks post-BDL. However, we cannot exclude that alternative substrates to Glc (such as Gln, ketone bodies, or fatty acids) may fuel the TCA cycle earlier in disease development, warranting further investigations.

The first-line antioxidants, Asc and GSH, maintain redox homeostasis and keep the balance between ROS generation and elimination (Pierzchala et al. 2022). A significant decrease in Asc concentration and a unique increase in GSH were observed in the cerebellum (Fig. 2), further highlighting this region as the one exhibiting the stronger neurometabolic changes. Our data showed brain redox homeostasis alterations in the cerebellum and corroborate our previous findings of direct ex-vivo detection of increased OS and significantly increased GPX-1 in the cerebellum of the BDL rat (Pierzchala et al. 2022) and previous post-mortem studies in patients^42^.

#### Striatum

The pattern of Gln increase in the striatum differed from that in other studied regions, together with smaller astrocytic alterations. In the cerebellum and hippocampus, Gln was consistently increasing over time, while in the striatum, Gln concentration increase started to decelerate at week-4. Interestingly, the main CNS osmolytes continued to strongly decrease after week-4 post-BDL, despite the lower Gln-induced osmotic pressure compared to the cerebellum. Tau showed a strong and significant decrease starting from week-4 post-BDL (Fig. 1B). This behavior may represent a delayed decrease consistent with osmolyte regulation or suggest that Tau assumes a different or an additional role (antioxidant/anti-inflammatory) to the osmoregulatory one (Ramírez-Guerrero et al. 2022) (supported by a significant correlation of Tau with NH_4_^+^ but not with Gln, Fig. S5). Tau plays a role in striatal plasticity, exerting a neuroprotective role under metabolic stress (Sergeeva et al. 2007). Therefore, substantial alterations in Tau may have molecular implications in this brain region. tCho showed the strongest and significantly different decrease in the striatum compared to the other brain regions. Apart from its osmoregulatory role, tCho is required for membrane phospholipid synthesis (Zeisel et al. 1986) and myelination, therefore, its strong decrease could be considered as a sign of neurodegeneration.

One limitation of the present study is the absence of behavioral assessments. Nonetheless, previous work from our group demonstrated that BDL rats exhibit reduced locomotor activity compared to sham-operated controls (Braissant et al. 2019). Consistent with these findings, other studies have reported impairments in spontaneous locomotion, exploratory behavior, learning, and memory in BDL rats (Leke et al. 2012, 2013; DeMorrow et al. 2021; Arenas et al. 2024), although no significant differences were observed in tasks such as the elevated plus-maze or foot-fault test (Leke et al. 2012). The mechanisms underlying these behavioral alterations remain poorly understood, including the specific brain regions and neurochemical pathways involved. It is important to note that even simple sensory, motor, and cognitive functions rely on the coordinated activity of multiple brain regions, complicating the interpretation of behavioral outcomes in relation to localized brain changes. In this context, the current study provides clear evidence of region-specific brain alterations in type C HE using in-vivo ^1^H-MRS, offering a valuable alternative to behavioral tests. Furthermore, the observed region-specific neurometabolic and cellular alterations identified in this study likely underline the behavioral dysfunctions observed in type C HE. These findings lay a critical foundation for future research aimed at establishing causal—rather than correlative-links with clinical symptomatology in HE.

## Conclusion

In an established model of type C HE, we identified both common and differential metabolic and cellular changes across brain regions. Common metabolic changes included elevation of Gln-chronologically first, decreases in osmolytes, and a decrease in neurotransmitters to varying degrees. At the cellular level, glial changes included an increase in astrocyte number, a decrease in number and length of astrocytic processes while microglia displayed increased cell perimeter and processes length, and an overall rise in number of microglia and amoeboid cells. Importantly, neurons showed a decreased spine density and soma size change (decreased soma size of Pj cells in the cerebellum vs. increased DG and CA1 neurons in the hippocampus). Further, each anatomical structure studied was characterized by its own metabolic and cellular signature. The cerebellum displayed the highest Gln burden, together with significant elevation of Lac and decreased GABA, as well as changes in antioxidants concentration. This was paralleled by pronounced astrocytic changes and decreased soma size of Pj cells, arguably related to the osmotic stress driven by Gln load. Next, the striatum displayed the lowest increase in brain Gln but the strongest decrease in brain osmolytes, potentially underlying the milder changes in astrocyte morphology. Taken together, our results highlight that although there are some common metabolic and cellular responses in type C HE, brain regions show a differential susceptibility, possibly related to cellular composition, although the teleological significance is unclear.

Based on the early rise of Gln in all brain regions studied, it is plausible that Gln is the molecular contributor to a cascade of cellular changes through osmotic and oxidative stress and neuroinflammation. Moreover, the observed neuronal alterations suggest an interdependence between astrocytic and neuronal morphological changes, which will affect the entire neuronal network activity and compromise brain function during disease progression, worsening neurological performances.

## Supplementary Information

Below is the link to the electronic supplementary material.

Supplementary Material 1 (DOCX 6.70 MB)

## Data Availability

The datasets generated during and/or analyzed during the current study are available from the corresponding author on reasonable request.
