# Supplementary material for: Differential metabolic and cellular brain regional susceptibility in adult rats with chronic liver disease
Source: Metab Brain Dis. 2026 Sep 11;41(1):215. doi: 10.1007/s11011-026-01954-1 (PMC13569521; doi:10.1007/s11011-026-01954-1)
Supplement: Supplementary file 1 [file 11011_2026_1954_MOESM1_ESM.docx]

# Differential metabolic and cellular brain regional vulnerability in adult rats with chronic liver disease

# Dunja Simicic, Katarzyna Pierzchala, Olivier Braissant, Dario Sessa, Valérie A. McLin, Cristina Cudalbu

# **Table of contents**

# **Supplementary Materials and Methods ........................................................................................ 2**

# **Supplementary Results ...................................................................................................................11**

# **Supplementary References .............................................................................................................24**

# **Supplementary Materials and Methods**

*BDL rat model of CLD induced-type C HE*

All experiments were approved by the Committee on Animal Experimentation for the Canton de Vaud, Switzerland (VD3022/VD2439), were conform to the Animal Research: Reporting of In Vivo Experiments (ARRIVE) guidelines (<http://www.nc3rs.org.uk/arrive-guidelines>) and all animals received human care. Wistar male adult rats (n=41, 175-200g, Charles River Laboratories, L’Arbresle, France) were used: 32 rats underwent BDL surgery, a model of type C HE accepted by ISHEN^1^, while the rest was Sham operated (BDL surgery protocol: <https://doi.org/10.5281/zenodo.10652104>). Animals were housed in cages (2-3 rats per cage) in the animal facility at 20–24° C with a 12 h light/dark cycle, and free access to water and food (SAFE 150 SP-25, SAFE, U8409G10R). Information about the number of animals for each experiment is provided in **Tables S1-S3**. Experiments were performed using isoflurane anesthesia (Piramal Pharma Ltd) (≈ 1.5%, in a 50%/50% air/O_2_ mix).

*Biochemical measurements to validate the CLD*

Liver function parameters (plasma bilirubin, aspartate aminotransferase (AST/GOT), alanine aminotransferase (ALT/GPT)), and glucose (Glc), were quantified using the Reflotron® Plus system (F. Hoffmann-La Roche Ltd.). Blood ammonia (NH₄⁺) concentrations were measured with the PocketChem™ BA PA-4140 analyzer. All parameters were assessed longitudinally at week 0 (before BDL surgery) and subsequently at 2-week intervals (weeks 2, 4, 6, and 8), as detailed in Supplementary **Table S1**.

### *In-vivo metabolic changes using ^1^H-MRS*

^1^H-MRS spectra were acquired on the 9.4 T system (Varian/Magnex Scientific) using the SPECIAL sequence (TE=2.8ms) as previously described^2^. Three different volumes of interest (VOI) were selected in hippocampus (2x2.8x2mm^3^), cerebellum (2.5x2.5x2.5mm^3^) and striatum (2.5x2x2.5mm^3^). LCModel was used for quantification with water as internal reference allowing the quantification of a total of 18 metabolites. The scans were performed before (week 0) and after BDL at weeks-2, 4, 6, and 8, thus each animal was its own control. For ^1^H-MRS from week-0 to week-6, 8 to 23 rats per week were scanned while at week-8 the number of scanned rats varied between the regions (cerebellum n=8, hippocampus n=7, and striatum n=4) (**Table S2**). Animals were sacrificed at different time points for histology measurements or because they reached the end point allowed by the Committee on Animal Experimentation for Canton of Vaud (e.g. 15% of weight loss), justifying the different number of rats at each time point. Importantly, all MRS measurements reported in this study, including those acquired from the hippocampus, were conducted on a distinct cohort of rats, separate from those previously described in our earlier publication^2^.

*Cellular morphological changes using histology*

**Tissue Preparation:** Animals were deeply anesthetized with 4% isoflurane (Piramal Pharma Ltd) for 5 min and an analgesic was injected (Temgesic (ESSEX) (0.1 mg/kg)) before transcardial perfusion with cell culture medium RPMI 1640 (R8758, pH 7.4, Sigma-Aldrich), supplemented with 10% of Fetal Bovine Serum (F7524, FBS, Sigma-Aldrich) and 1% of antibiotics (P4083, 50.5 units/ml penicillin, 50.5 μg/ml streptomycin and 101 μg/ml neomycin, Sigma) to wash out blood and keep the brain cells alive, pH 7.4.

**Immunohistochemistry (IHC):** Animals were sacrificed for histological evaluation at week-4 (BDL, n=3) and 8 (BDL, n=3 and Sham, n=3) post-surgery (**Table S3**). Brains were removed and fixed in 4% formaldehyde PBS solution overnight at 4°C, followed by PBS wash and 48h cryopreservation in 30% sucrose in PBS at 4°C. They were embedded in an optimum cutting temperature compound (4583, Tissue-Tek® O.C.T. Compound, SAKURA) then flash-frozen using dry ice and 2-Methylbutane (M32631, Sigma-Aldrich) for sectioning. Sections were mounted on Superfrost Plus microscope slides (22037246, Thermo Scientific). After the staining procedure, the slides were mounted with ProLong™ Diamond Antifade Mountant (P36970, Thermo Fisher) and covered with coverslips.

**Fluorescence microscopy** examination of tissue sections was performed. Rat brains (week-4 post-BDL (n=3), week-8 post-BDL (n=3) and Sham (n=3), **Table S3**) were sliced into 16 μm thick sagittal sections using a Leica Jung CM 1800 cryostat. For each rat, seven different slides were analyzed (distance between tissue sections ~250μm), resulting in a total of 63 slides prepared. To investigate the morphometrical changes of astrocytes and microglia in the hippocampus, cerebellum, and striatum, the Sholl analysis was implemented (**Fig. S1**)^2^. For the astrocytes, each process was separated by segment order – concentric spheres originating from the soma (**Fig. S1A**). The number of processes leaving the cell body, total length, and intersections of processes were quantified and plotted according to subregions (1-3) (**Fig. S1A**). The microglia (**Fig. S1B**) cell body perimeter was determined by encircling the microglia using the polygon tool to connect distal extremities of every process. The number of primary processes and total length of all processes were measured.

The number of data points differs between measures because the number of analyzable ROIs per section depends on the cutting plane and staining quality. To minimize quantification errors due to overlapping cells, only uniformly stained cells with clearly separated processes were included for astrocyte intersection (Sholl ring) analysis and microglial perimeter analysis. Processes length analysis was performed on uniformly stained, continuous processes (same focus plane) of astrocytes and microglia.

Integer numbers of astrocytes, microglia, and amoeboid microglia were counted within defined larger regions of interest (ROIs) and subsequently normalized to a standard surface area of 0.01 mm².

In line with the 3R (Replacement, Reduction, and Refinement) principles, a subgroup of rats (No. BDL 340, 468, 472, and Sham 388, 469, 470) used in the current study have been previously described and characterized in an earlier publication^4^, however, a range of additional histological measurements were added in the current study: 1) For hippocampal astrocytes we characterized the no. of intersections (Rings 1, 2, 3) at week 8; 2) cerebellar and striatal astrocytes were also characterized at week 8. Furthermore, a longitudinal characterization of astrocytes at week 4 was conducted on additional animals (see Table S3).

**Fig. S1.** **Sholl analysis:** **(A)** Astrocytes - morphological characterization of the number of intersections of branches with radii at various distances from the cell body. **(B)** Microglia - morphological characterization of the cell perimeter, and **(C)** Amoeboid microglia are distinguished as big, spherical stained cells with few, short or no processes.

**Golgi**-Cox **staining**^3,5^ was performed to unveil the detailed morphology of the hippocampus CA1 neurons and DG granule cells (**Fig.** S2) and cerebellar Pj cells. Brains (week-8 BDL (n=6) and Sham (n=6), **Table S3**) were cut into 115 μm thick sagittal sections using Leica VT1200 S vibratome. Tissues with homogeneous staining and clearly visible dendritic segments and spines were chosen for quantitative analysis (25 slides/hemisphere). The surface of the neuronal soma was measured, and manual counting of the dendrite spine was conducted^6^. Measurements were obtained from the CA1 neurons (BDL soma ~200 cells, apical and basal dendrites ~100 each; Sham soma ~120 cells, apical and basal dendrites ~60 each) and DG granule cells (BDL soma ~200 cells, apical dendrites ~150; Sham soma ~180 cells, apical dendrites ~80) of the hippocampus and Pj cells (BDL soma ~500 cells, apical dendrites ~220; Sham soma ~180 cells, apical dendrites ~80) in the cerebellum. In line with the 3R (Replacement, Reduction, and Refinement) principles, a subgroup of rats (No. BDL 355, 356, 365, 368 and Sham 360, 363) used in the current study have been previously described and characterized in earlier publications^4,6^; however, a range of additional histological measurements were added in the current study: 1) Characterization of hippocampal dentate gyrus neurons and 2) Characterization of cerebellar Pj cells.

**Fig. S2. Representative micrograph of Golgi–Cox impregnated hippocampus** (scale bar = 200 μm).

Sections (IHC and Golgi Cox) were digitized using a MEIJI TECHNO TC5600 microscope with an INFINITYX-32 camera. To provide comprehensive analysis, images were captured using 5x, 10x, 20x, and 50x objectives. Images were acquired and analyzed using the INFINITY ANALYZE 7 software (Lumenera, Canada).

*Statistical analysis*

**^1^H-MRS** data are displayed as scatter plots showing the relative increase of metabolite concentrations over time during disease progression. The plotted values were obtained by subtracting the week 0 concentration of a metabolite for every rat and brain region from the corresponding week 2, 4, 6 and 8 measured concentrations and therefore keeping only the relative (to week 0) change in the disease. In this way the starting differences in metabolite concentrations between the brain regions^7,8^ were removed, describing only the metabolite evolution due to the disease while clearly pointing to the brain regional difference for the concerned metabolites. The plots were created in RStudio^9^ where additionally a LOWESS (Locally Weighted Scatterplot Smoothing) regression was applied and plotted to better visualize and distinguish data trends between brain regions.

**STATIS:** STATIS method is created as an extension to PCA, designed to manage multiple data tables that contain set of variables collected on the same observations^12,13^.

As described, the animals were scanned (in three brain regions), and blood sampling was performed longitudinally at weeks 0, 2, 4, 6, and 8. For one brain region this provides us with several quantified metabolites for each rat at five different time points. Therefore, we have a three-dimensional dataset suitable for the STATIS analysis. The analysis was performed on each brain region individually on a combined dataset consisting of the brain metabolites and blood parameters.

The data was prepared by stacking rats at different time points as rows and metabolites as columns (for each brain region). This resulted in three datasets: brain metabolites + blood parameters – striatum, brain metabolites + blood parameters – hippocampus, and brain metabolites + blood parameters – cerebellum. Every dataset was processed using the following procedure. The matrix/dataset (rats at different time points -rows, metabolites -columns) was centered by subtracting the mean of every column to the corresponding column (e.g. the mean value of Gln measured for all the rats at all time points was subtracted from every Gln measurement). The few missing values due to low spectral quality or missing a scan were imputed using the missMDA package (RStudio).

**Correlations:** To test the correlations between the longitudinally acquired brain metabolites with brain Gln and blood NH4+ values (metabolites considered responsible for many of the changes in HE), Pearson correlation analysis was performed.

**Table S1. Detailed report on each rat used for biochemical measures.**

|  | **Blood (**$\mathbf{NH}_{\mathbf{4}}^{\mathbf{+}}$**) and Plasma (GOT, GPT, Bilirubin) - P** | | | | | | | | | |
| --- | --- | --- | --- | --- | --- | --- | --- | --- | --- | --- |
|  | **week-0** | | **week-2** | | **week-4** | | **week-6** | | **week-8** | |
|  | **B** | **P** | **B** | **P** | **B** | **P** | **B** | **P** | **B** | **P** |
| **BDL 200** |  |  |  |  |  |  |  |  |  |  |
| **BDL 211** |  |  |  |  |  |  |  |  |  |  |
| **BDL 213** |  |  |  |  |  |  |  |  |  |  |
| **BDL 326** |  |  |  |  |  |  |  |  |  |  |
| **BDL 340** |  |  |  | x |  | x |  | x |  | x |
| **BDL 341** |  |  |  | x |  | x |  | x |  | x |
| **BDL 354** |  | x |  |  |  | x |  | x |  |  |
| **BDL 355** |  |  |  |  |  |  |  |  |  |  |
| **BDL 356** |  |  |  |  |  |  |  |  |  |  |
| **BDL 357** |  |  |  |  |  |  |  |  |  |  |
| **Sham 358** |  |  |  |  |  |  |  |  |  |  |
| **Sham 359** |  |  |  |  |  |  |  |  |  |  |
| **Sham 360** |  |  |  |  |  |  |  |  |  |  |
| **Sham 361** |  |  |  |  |  |  |  |  |  |  |
| **Sham 362** |  |  |  |  |  |  |  |  |  |  |
| **Sham 363** |  |  |  |  |  |  |  |  |  |  |
| **BDL 365** |  |  |  |  |  |  |  |  |  |  |
| **BDL 367** |  |  |  |  |  |  |  |  |  |  |
| **BDL 368** |  |  |  |  |  |  |  |  |  |  |
| **BDL 385** |  | x |  | x |  | x |  | x |  |  |
| **Sham 388** |  |  |  |  |  |  |  |  |  |  |
| **BDL 389** |  | x |  | x |  | x |  | x |  |  |
| **BDL 404** | x | x | x | x | x | x |  |  |  |  |
| **BDL 405** | x | x | x | x |  |  |  |  |  |  |
| **BDL 406** | x | x | x | x |  |  |  |  |  |  |
| **BDL 407** | x | x | x | x | x | x | x | x |  |  |
| **BDL 412** | x | x | x | x | x | x | x | x |  |  |
| **BDL 413** | x | x | x | x | x | x | x | x |  |  |
| **BDL 414** | x | x | x | x | x | x | x | x | x | x |
| **BDL 415** | x | x | x | x | x | x | x | x | x | x |
| **BDL 466** | x | x | x | x | x | x | x | x |  |  |
| **BDL 467** | x | x | x | x | x | x | x | x |  |  |
| **BDL 468** | x | x | x | x | x | x | x | x | x | x |
| **Sham 469** |  |  |  |  |  |  |  |  |  |  |
| **Sham 470** |  |  |  |  |  |  |  |  |  |  |
| **BDL 471** | x | x | x | x | x | x | x | x |  |  |
| **BDL 472** | x | x | x | x | x | x | x | x | x | x |
| **BDL 474** | x | x | x | x | x | x | x | x |  |  |
| **BDL 475** | x | x |  | x | x | x |  |  |  |  |
| **BDL 476** |  |  |  |  |  |  |  |  |  |  |
| **BDL 478** |  |  |  |  |  |  |  |  |  |  |
| **N = 41** | **n = 15** | **n = 18** | **n = 14** | **n = 19** | **n = 13** | **n = 18** | **n = 10** | **n = 15** | **n = 4** | **n = 6** |

**Table S2. Detailed report on each rat used in the 1H-MRS experiment.** Number of animals is different per time point due to scanner time limitations and sacrifices for histological measures.

|  | **^1^H-MRS cerebellum** | | | | | **^1^H-MRS hippocampus** | | | | | **^1^H-MRS striatum** | | | | |
| --- | --- | --- | --- | --- | --- | --- | --- | --- | --- | --- | --- | --- | --- | --- | --- |
|  | **Week** | | | | | **week** | | | | | **week** | | | | |
|  | **0** | **2** | **4** | **6** | **8** | **0** | **2** | **4** | **6** | **8** | **0** | **2** | **4** | **6** | **8** |
| **BDL 200** | x |  | x | x | x | x |  | x | x | x |  |  |  |  |  |
| **BDL 211** | x | x | x | x | x | x | x | x | x | x |  |  |  |  |  |
| **BDL 213** | x | x | x | x | x | x | x | x | x | x |  |  |  |  |  |
| **BDL 326** | x |  |  |  | x |  |  |  |  |  |  |  |  |  |  |
| **BDL 340** | x |  | x | x |  | x |  | x | x |  |  |  |  |  |  |
| **BDL 341** | x |  | x | x |  | x |  | x | x |  |  |  |  |  |  |
| **BDL 354** | x |  | x | x |  | x |  | x | x |  |  |  |  |  |  |
| **BDL 355** |  |  |  | x |  |  |  |  |  |  |  |  |  |  |  |
| **BDL 356** |  |  |  | x |  |  |  |  |  |  |  |  |  |  |  |
| **BDL 357** |  |  |  | x |  |  |  |  |  |  |  |  |  |  |  |
| **BDL 365** |  |  |  |  |  |  |  |  |  |  |  |  |  |  |  |
| **BDL 367** |  |  |  |  |  |  |  |  |  |  |  |  |  |  |  |
| **BDL 368** |  |  |  |  |  |  |  |  |  |  |  |  |  |  |  |
| **BDL 385** | x |  | x | x |  | x | x |  |  |  |  |  |  |  |  |
| **BDL 389** | x |  | x | x |  | x |  | x | x |  |  |  |  |  |  |
| **BDL 404** | x | x | x |  |  | x | x | X |  |  | x | x | x |  |  |
| **BDL 405** | x | x |  |  |  | x | x |  |  |  | x | x |  |  |  |
| **BDL 406** | x | x |  |  |  | x | x |  |  |  | x | x |  |  |  |
| **BDL 407** | x | x | x |  |  | x | x | x |  |  | x | x | x |  |  |
| **BDL 412** | x | x | x | x |  | x | x | x | x |  | x | x | x | x |  |
| **BDL 413** | x | x | x | x |  | x | x | x | x |  | x | x | x | x |  |
| **BDL 414** | x | x | x | x | x | x | x | x | x | x | x | x | x | x | x |
| **BDL 415** | x |  | x | x | x | x |  | x | x | x | x |  | x | x | x |
| **BDL 466** | x | x | x | x |  | x | x | x | x |  | x | x | x | x |  |
| **BDL 467** | x | x | x | x |  | x | x | x | x |  | x | x | x | x |  |
| **BDL 468** | x | x | x | x | x | x | x | x | x | x | x | x | x | x | x |
| **BDL 471** | x | x | x | x |  | x | x | x | x |  | x | x | x | x |  |
| **BDL 472** | x | x | x | x | x | x | x | x | x | x | x | x | x | x | x |
| **BDL 474** |  | x | x | x |  |  | x | x | x |  |  | x | x | x |  |
| **BDL 475** |  |  |  |  |  |  |  |  |  |  |  |  |  |  |  |
| **BDL 476** |  |  |  |  |  |  |  |  |  |  |  |  |  |  |  |
| **BDL 478** |  |  |  |  |  |  |  |  |  |  |  |  |  |  |  |
| **N = 32** | **n = 23** | **n = 15** | **n = 21** | **n = 21** | **n = 8** | **n = 22** | **n = 15** | **n = 21** | **n = 18** | **n = 7** | **n = 15** | **n = 14** | **n = 13** | **n = 10** | **n = 4** |

**Table S3. Detailed report on each rat used for histological measures.**

|  | **Golgi Cox staining** | | **GFAP staining** | | | **Iba-1 staining** | | |
| --- | --- | --- | --- | --- | --- | --- | --- | --- |
|  | **week-8** | **control** | **week-4** | **week-8** | **control** | **week-4** | **week-8** | **control** |
| **BDL 200** |  |  |  |  |  |  |  |  |
| **BDL 211** |  |  |  |  |  |  |  |  |
| **BDL 213** |  |  |  |  |  |  |  |  |
| **BDL 326** |  |  |  |  |  |  |  |  |
| **BDL 340** |  |  |  | x |  |  | x |  |
| **BDL 341** |  |  |  |  |  |  |  |  |
| **BDL 354** |  |  |  |  |  |  |  |  |
| **BDL 355** | x |  |  |  |  |  |  |  |
| **BDL 356** | x |  |  |  |  |  |  |  |
| **BDL 357** | x |  |  |  |  |  |  |  |
| **Sham 358** |  | x |  |  |  |  |  |  |
| **Sham 359** |  | x |  |  |  |  |  |  |
| **Sham 360** |  | x |  |  |  |  |  |  |
| **Sham 361** |  | x |  |  |  |  |  |  |
| **Sham 362** |  | x |  |  |  |  |  |  |
| **Sham 363** |  | x |  |  |  |  |  |  |
| **BDL 365** | x |  |  |  |  |  |  |  |
| **BDL 367** | x |  |  |  |  |  |  |  |
| **BDL 368** | x |  |  |  |  |  |  |  |
| **BDL 385** |  |  |  |  |  |  |  |  |
| **Sham 388** |  |  |  |  | x |  |  | x |
| **BDL 389** |  |  |  |  |  |  |  |  |
| **BDL 404** |  |  |  |  |  |  |  |  |
| **BDL 405** |  |  |  |  |  |  |  |  |
| **BDL 406** |  |  |  |  |  |  |  |  |
| **BDL 407** |  |  |  |  |  |  |  |  |
| **BDL 412** |  |  |  |  |  |  |  |  |
| **BDL 413** |  |  |  |  |  |  |  |  |
| **BDL 414** |  |  |  |  |  |  |  |  |
| **BDL 415** |  |  |  |  |  |  |  |  |
| **BDL 466** |  |  |  |  |  |  |  |  |
| **BDL 467** |  |  |  |  |  |  |  |  |
| **BDL 468** |  |  |  | x |  |  | x |  |
| **Sham 469** |  |  |  |  | x |  |  | x |
| **Sham 470** |  |  |  |  | x |  |  | x |
| **BDL 471** |  |  |  |  |  |  |  |  |
| **BDL 472** |  |  |  | x |  |  | x |  |
| **BDL 474** |  |  |  |  |  |  |  |  |
| **BDL 475** |  |  | x |  |  | x |  |  |
| **BDL 476** |  |  | x |  |  | x |  |  |
| **BDL 478** |  |  | x |  |  | x |  |  |
| **N = 41** | **n = 6** | **n = 6** | **n = 3** | **n = 3** | **n = 3** | **n = 3** | **n = 3** | **n = 3** |

# **Supplementary Results**

### BDL animals showed increased blood bilirubin and ammonium from week-2, validating the development of CLD (**Fig.S3**) validating the presence of CLD.

**Fig. S3. Longitudinal changes in total plasma bilirubin,** **aspartate aminotransferase (AST/GOT) and alanine aminotransferase (ALT/GPT); and blood NH_4_^+^ and glucose (Glc) induced by bile duct ligation.** Bilirubin was non-measurable before BDL. Significance level between week 0 and weeks 2-8: *p<0.05, **p<0.01, ***p<0.001, ****p<0.0001 (1-way ANOVA), mean ± SD.

**Fig. S4:** **Relative changes of glutamine and CNS osmolytes in the three brain regions.** The scatter plots display the relative increase of metabolite concentrations (for Gln, Ins, tCho - GPC.PCho, Tau, Cr and tCr – Cr.PCr) over time during the disease progression.

**Fig. S5: Pearson correlations between brain metabolites (Gln, Ins, tCho, Tau and Cr) and blood NH_4_^+^** throughout the full course of the study for the striatum, hippocampus, and cerebellum. The corresponding *p* values are shown along with the Pearson correlation coefficients. Statistical significance: *p<0.05, **p<0.01, ***p<0.001, ****p<0.0001.

**Fig. S6.** **Relative changes of other main brain metabolites in the three brain regions**. The scatter plots display the relative increase of metabolic concentrations (for Glu, GABA, NAA, Lac, Asc, and GSH) over time during the disease progression.

**Fig. S7. Pearson correlations between brain metabolites (for Glu, GABA, NAA, Lac, Asc and GSH) and blood NH_4_^+^** throughout the full course of the study for striatum, hippocampus and cerebellum. The corresponding *p* values are shown along with the Pearson correlation coefficients. Statistical significance: *p<0.05, **p<0.01, ***p<0.001, ****p<0.0001.

**Table S4. Results of morphometry measurements of astrocytes (GFAP) and microglia (Iba1) (Sholl analysis) for all the studied brain regions.** Presented as mean ± SD along with the corresponding relative percentage changes. One-way Anova with post-hoc Tukey HSD, *p<0.05, **p<0.01, ***p<0.001, ****p<0.0001, mean ± SD.

|  | ***Astrocytes*** | | | | | | ***Microglia*** | | | | ***All*** |
| --- | --- | --- | --- | --- | --- | --- | --- | --- | --- | --- | --- |
| ***Hippocampus*** | ***Number of processes*** | ***Processes length [µm]*** | ***Processes intersections*** | | | ***Astrocytes***  ***cells / 0.01 mm^2^*** | ***Number of processes*** | ***Processes length [µm]*** | ***Perimeter [µm]*** | ***Microglia***  ***cells / 0.01 mm^2^*** | ***Total***  ***nucleus / 0.01 mm^2^*** |
|  |  |  | ***Ring 1*** | ***Ring 2*** | ***Ring 3*** |  |  |  |  |  |  |
| ***Sham*** | 8.24 ± 1.4 | 34.02 ± 6.5 | 20.88 ± 3.3 | 23 ± 5.3 | 12.5 ± 5.5 | 4.27 ± 0.9 | 4.89 ± 1.13 | 37.2 ± 7.5 | 187.17 ± 25.9 | 1.0 ± 0.41 | 14.51 ± 3.3 |
| ***BDL w4*** | 7.76 ± 1.2 | 29.51 ± 6.8 | 19.71 ± 3.6 | 16.94 ± 2.9 | 6.94 ± 2.7 | 6.29 ± 1.8 | 4.69 ± 0.9 | 44.1 ± 9.43 | 205.93 ± 32 | 1.82 ± 0.44 | 21.07 ± 5.2 |
| ***BDL w8*** | 6.69 ± 1.1 | 22.99 ± 4.9 | 17.85 ± 3.1 | 14.02 ± 4.6 | 3.44 ± 2.7 | 4.61 ± 0.4 | 4.64 ± 1.03 | 39.52 ± 7.69 | 204.13 ± 25.64 | 1.31 ± 0.44 | 18.36 ± 3.2 |
| ***Relative change [%]*** | | | | | | | | | | | |
| ***Sham vs. BDL w4*** | - 5.79*** | -13.26**** | - 5.60 | -26.34**** | - 44.47**** | + 47.48*** | - 4.12 | + 18.57** | + 10.03** | + 39.9** | + 45.26** |
| ***Sham vs. BDL w8*** | - 18.77**** | -32.41**** | - 14.47** | - 39.02**** | - 72.49**** | + 8.09 | - 5.03 | + 6.25** | + 9.07** | + 0.07 | + 26.57* |
| ***BDL w4 vs. BDL w8*** | - 13.78**** | -22.08**** | - 9. 4* | - 17.22** | - 50.46**** | - 26.71** | - 0.95 | - 10.39** | - 0.87 | - 27.99** | - 12.87 |

|  | ***Astrocytes*** | | | | | | ***Microglia*** | | | | ***All*** |
| --- | --- | --- | --- | --- | --- | --- | --- | --- | --- | --- | --- |
| ***Cerebellum*** | ***Number of processes*** | ***Processes length [µm]*** | ***Processes intersections*** | | | ***Astrocytes***  ***cells / 0.01 mm^2^*** | ***Number of processes*** | ***Processes length [µm]*** | ***Perimeter [µm]*** | ***Microglia***  ***cells / 0.01 mm^2^*** | ***Total***  ***nucleus / 0.01 mm^2^*** |
|  |  |  | ***Ring 1*** | ***Ring 2*** | ***Ring 3*** |  |  |  |  |  |  |
| ***Sham*** | 11.44 ± 1.6 | 35.66 ± 8.2 | 16.61 ± 2.4 | 14.76 ± 4.7 | 6.82 ± 3.4 | 3.35 ± 0.6 | 4.21 ± 0.97 | 37.38 ± 9.2 | 169.0 ± 18.9 | 1.03 ± 0.36 | 198.24 ± 24.1 |
| ***BDL w4*** | 9.13 ± 1.99 | 29.78 ± 7.4 | 14.38 ± 2.37 | 13.54 ± 3.1 | 6.72 ± 3.4 | 4.86 ± 0.8 | 3.7 ± 0.68 | 47.78 ± 10.8 | 200.25 ± 35.5 | 1.31 ± 0.39 | 303.86 ± 15.8 |
| ***BDL w8*** | 6.8 ± 1.41 | 24.1 ± 5.81 | 13.18 ± 1.9 | 10.56 ± 2.43 | 3.36 ± 1.8 | 4.11 ± 0.6 | 4.74 ± 0.99 | 41.23 ± 8.3 | 205.89 ± 24.9 | 1.06 ± 0.34 | 239.78 ± 34.2 |
| ***Relative change [%]*** | | | | | | | | | | | |
| ***Sham vs. BDL w4*** | - 20.15** | -16.48** | - 13.4** | - 8.28 | - 1.26 | + 45.18*** | - 12.2* | + 27.82** | + 18.49** | + 27.24* | + 53.29*** |
| ***Sham vs. BDL w8*** | - 40.52** | -32.43** | - 20.6** | - 28.25** | - 50.6** | + 22.72 | + 5.76 | + 9.33** | +17.92** | + 2.53 | + 20.96** |
| ***BDL w4 vs. BDL w8*** | - 25.52** | -19.09** | - 8.3* | - 21.8** | - 49.96** | - 15.47** | + 20.87** | - 13.729** | +2.82 | - 19.36* | - 21.09*** |

|  | ***Astrocytes*** | | | | | | ***Microglia*** | | | | ***All*** |
| --- | --- | --- | --- | --- | --- | --- | --- | --- | --- | --- | --- |
| ***Striatum*** | ***Number of processes*** | ***Processes length [µm]*** | ***Processes intersections*** | | | ***Astrocytes***  ***cells / 0.01 mm^2^*** | ***Number of processes*** | ***Processes length [µm]*** | ***Perimeter [µm]*** | ***Microglia***  ***cells / 0.01 mm^2^*** | ***Total***  ***nucleus / 0.01 mm^2^*** |
|  |  |  | ***Ring 1*** | ***Ring 2*** | ***Ring 3*** |  |  |  |  |  |  |
| ***Sham*** | 8.74 ± 1.2 | 37.71 ± 7.9 | 15.81 ± 3.5 | 14.55 ± 5.1 | 6.09 ± 2.3 | 4.18 0.9 | 5.04 ± 0.83 | 35.79 ± 7.67 | 183.14 ± 22.74 | 1.23 ± 0.35 | 32.45 ± 2.9 |
| ***BDL w4*** | 8.31 ± 1.6 | 31.05 ± 5.6 | 16.44 ± 3.1 | 11.89 ± 2.7 | 4.67 ± 2.3 | 5.46 ± 0.7 | 4.69 ± 1.01 | 41.51 ± 9.22 | 201.14 ± 19.36 | 1.7 ± 0.6 | 39.14 ± 5.1 |
| ***BDL w8*** | 7.71 ± 1.2 | 30.14 ± 6.1 | 16.92 ± 4.3 | 12.91 ± 4.5 | 5.17 ± 2.4 | 4.81 ± 0.5 | 4.76 ± 1.06 | 38.07 ± 8.26 | 198.42 ± 25.88 | 1.38 ± 0.42 | 41.6 ± 5.7 |
| ***Relative change [%]*** | | | | | | | | | | | |
| ***Sham vs. BDL w4*** | - 4.9 | -17.64*** | + 4 | - 18.3 | - 23.4 | + 30.76*** | - 1.44 | + 15.95** | + 9.83** | + 38.6** | + 20.6*** |
| ***Sham vs. BDL w8*** | - 11.76** | -20.1*** | + 6.9 | - 11.2 | - 15.2 | + 15.2 | - 5.54 | + 6.36* | + 8.35** | + 12.3 | + 28.12*** |
| ***BDL w4 vs. BDL w8*** | - 7.2 | -2.94 | + 2.9 | + 8.6 | + 10.7 | - 11.93* | - 4.15 | - 8.27** | - 1.35 | - 18.96 | + 6.24 |

|  | ***Cells / 0.01 mm^2^*** | | |
| --- | --- | --- | --- |
| ***Amoeboid microglia*** | ***Cerebellum*** | ***Hippocampus*** | ***Striatum*** |
| ***Sham*** | —------------- | —------------- | —------------- |
| ***BDL w4*** | 0.33 ± 0.23 | 0.20 ± 0.18 | 0.21 ± 0.30 |
| ***BDL w8*** | 0.67 ± 0.37 | 0.50 ± 0.35 | 0.67 ± 0.58 |
| ***Relative change [%]*** | | | |
| ***BDL w4 vs. BDL w8*** | 102** | 150* | 212* |

**GFAP:** mean ± SD per animal

| ***Hippocampus*** | ***Number of*** ***astrocytes / 0.01 mm^2^*** | ***Number of processes*** | ***Processes length [µm]*** | ***Ring 1*** | ***Ring 2*** | ***Ring 3*** |
| --- | --- | --- | --- | --- | --- | --- |
| SHAM 388 | 4.4 ± 0.7 | 8.6 ± 1.4 | 33.1 ± 6.5 | 20.3 ± 0.9 | 16 ± 3.1 | 8.3 ± 3.3 |
| SHAM 469 | 3.8 ± 1 | 8.1 ± 1.4 | 33.7 ± 6.1 | 21.1 ± 4.4 | 24.9 ± 3.9 | 14 ± 6.4 |
| SHAM 470 | 4.9 ± 0.3 | 7.5 ± 1.3 | 35.9 ± 7.1 | 21 ± 1 | 26.7 ± 2.1 | 15.3 ± 2.5 |
|  | | | | | | |
| BDL 475 w4 | 5.4 ± 0.7 | 7.5 ± 1.3 | 25.1 ± 4.7 | 18.8 ± 4.7 | 16 ± 4.2 | 6 ± 2 |
| BDL 476 w4 | 6.1 ± 0.3 | 7.7 ± 1.3 | 28.7 ± 6.3 | 18.9 ± 3.4 | 16.7 ± 2.7 | 8.4 ±3 |
| BDL 478 w4 | 7.5 ± 3.4 | 7.8 ± 1.1 | 31.3 ± 3 | 20.7 ± 3.2 | 17.5 ± 2.4 | 6.2 ± 2.2 |
|  | | | | | | |
| BDL 340 w8 | 4.7 ± 0.6 | 6.6 ± 1.2 | 21.9 ± 4.3 | 18.3 ± 3.6 | 15 ± 6.7 | 4.3 ± 3.1 |
| BDL 468 w8 | 4.4 ± 0.3 | 6.6 ± 1 | 23.3 ± 4.8 | 18.6 ± 2.6 | 14.4 ± 4.7 | 3.3 ± 2.5 |
| BDL 472 w7 | 4.7 ± 0.3 | 6.9 ± 1 | 23.3 ± 5.2 | 16.3 ± 3.1 | 12.7 ± 2.6 | 3.2 ± 2.9 |

| ***Cerebellum*** | ***Number of*** ***astrocytes / 0.01 mm^2^*** | ***Number of processes*** | ***Processes length [µm]*** | ***Ring 1*** | ***Ring 2*** | ***Ring 3*** |
| --- | --- | --- | --- | --- | --- | --- |
| SHAM 388 | 3.2 ± 0.6 | 11.4±1.9 | 34.5±8.7 | 15.4 ±1.7 | 12.3± 3.1 | 5.4 ± 2.2 |
| SHAM 469 | 3.4 ± 0.8 | 11.5 ± 1.7 | 38 ±7.8 | 16.9 ± 2.4 | 15.9 ±3.7 | 7.7 ±3.2 |
| SHAM 470 | 3.5 ± 0.4 | 11.4 ± 1.4 | 35 ± 7.6 | 18.1 ± 2.6 | 16.3 ± 7.5 | 7.1 ±5.2 |
|  | | | | | | |
| BDL 475 w4 | 4.3 ± 0.6 | 9.6 ± 1.5 | 29.6 ± 6.9 | 15 ± 2.9 | 13.8 ± 4.6 | 7.1 ± 4.5 |
| BDL 476 w4 | 4.8 ± 0.7 | 10.5 ± 2.2 | 31.3 ± 6.2 | 16 ± 2.4 | 15.9 ± 2.5 | 8.4 ± 3.6 |
| BDL 478 w4 | 5.8 ± 0.4 | 7.3 ± 1.2 | 28.6 ± 8.3 | 13.3 ± 1.5 | 12.3 ± 1.5 | 5.7 ± 2.4 |
|  | | | | | | |
| BDL 340 w8 | 4.7 ± 0.5 | 5.7 ± 1.4 | 24.6 ± 5.8 | 11 ± 1 | 8.7 ± 1.5 | 4.3 ± 0.6 |
| BDL 468 w8 | 4.4 ± 0.3 | 7.1 ± 1.3 | 23.7 ± 5.5 | 12.9 ± 1.8 | 10.3 ± 2.7 | 3.3 ± 2 |
| BDL 472 w7 | 4.7 ± 0.2 | 6.8 ± 1.4 | 24.7 ± 6.3 | 13.6 ± 1.8 | 11.5 ± 1.8 | 3.4 ± 1.5 |

| ***Striatum*** | ***Number of*** ***astrocytes / 0.01 mm^2^*** | ***Number of processes*** | ***Processes length [µm]*** | ***Ring 1*** | ***Ring 2*** | ***Ring 3*** |
| --- | --- | --- | --- | --- | --- | --- |
| SHAM 388 | 3.1 ± 0.4 | 8.8± 1.5 | 40.4± 11.2 | 15 ± 3 | 11.3 ± 1.5 | 4.3 ± 2.3 |
| SHAM 469 | 3.3 ± 0.9 | 8.2 ± 0.8 | 37.5 ± 6.4 | 16.3 ± 4.6 | 15.3 ± 4.3 | 7.8 ± 1.5 |
| SHAM 470 | 3.5 ± 0.5 | 9.1 ± 1.1 | 36.9 ± 8.1 | 16 ± 3.5 | 16.3 ± 7.1 | 5.8 ± 2.2 |
|  | | | | | | |
| BDL 475 w4 | 5.8 ± 0.6 | 8.3 ± 1.5 | 33.8 ± 4.9 | 16.6 ± 3.9 | 12.6 ± 3.3 | 4.8 ± 1.7 |
| BDL 476 w4 | 5.3 ± 1 | 7.5 ± 1.6 | 28.8 ± 5.3 | 17.3 ± 3.2 | 11 ± 2 | 1.3 ± 0.6 |
| BDL 478 w4 | 5.7 ± 0.8 | 8.6 ± 1.6 | 30.2 ± 5.6 | 15.8 ± 1.8 | 11.3 ± 2.1 | 6.1 ± 1.9 |
|  | | | | | | |
| BDL 340 w8 | 4.6 ± 0.2 | 7.3 ± 1.1 | 29.9 ± 6.3 | 16.2 ± 5.2 | 13.7 ± 4.3 | 5.2 ± 2.2 |
| BDL 468 w8 | 4.5 ± 0.1 | 7 ± 0.7 | 31.3 ± 5 | 13.7 ± 2.5 | 9 ± 1.7 | 3.3 ± 0.6 |
| BDL 472 w7 | 4.2 ± 0.6 | 8.4 ± 1.2 | 29.6 ± 6.4 | 19.2 ± 3.6 | 14.6 ±5 | 6.2 ± 2.8 |

| ***All cells DAPI*** | ***Cerebellum*** | ***Hippocampus*** | ***Striatum*** |
| --- | --- | --- | --- |
| SHAM 388 | 183.7 ± 42.4 | 15.1 ± 2.6 | 33.9 ± 1.2 |
| SHAM 469 | 206 ± 19.4 | 16.3 ± 4.6 | 32.2 ± 3.8 |
| SHAM 470 | 201.3 ± 7.5 | 12.2 ± 0.9 | 31.4 ± 2.5 |
|  | | | |
| BDL 475 w4 | 307.2 ± 17.7 | 23.8 ± 7.1 | 40.4 ± 1.2 |
| BDL 476 w4 | 298.8 ± 14.9 | 21.2 ± 3.9 | 36.2 ± 1 |
| BDL 478 w4 | 293 ± 26.8 | 18.9 ± 5 | 38.7 ± 5.4 |
|  | | | |
| BDL 340 w8 | 286.6 ± 2.4 | 19.9 ± 0.4 | 40.6 ± 0.9 |
| BDL 468 w8 | 236.8 ± 11.2 | 17.8 ± 4 | 35.3 ± 0.5 |
| BDL 472 w7 | 198.9 ± 4 | 16.9 ± 4.5 | 41.3 ±4.7 |

**Iba1:** mean ± SD per animal

| ***Hippocampus*** | ***Number of*** ***microglia / 0.01 mm^2^*** | ***Number of processes*** | ***Processes length [µm]*** | ***Perimeter [µm]*** | ***Amoeboid microglia / 0.01 mm^2^*** |
| --- | --- | --- | --- | --- | --- |
| SHAM 388 | 1.4 ± 0.5 | 4.3 ± 1.4 | 37.5 ± 6.9 | 188.3 ± 24.1 | - |
| SHAM 469 | 1 ± 0.3 | 5.2 ± 0.6 | 36.9 ± 7.8 | 187.8 ± 19.7 | - |
| SHAM 470 | 1.4 ± 0.3 | 5.8 ± 0.9 | 36.8 ± 8.5 | 183.6 ± 35.9 | - |
|  | | | | | |
| BDL 475 w4 | 2 ± 0.4 | 4.5 ± 0.8 | 42.5 ± 8.8 | 196.1 ± 32.8 | 0.2 ± 0.2 |
| BDL 476 w4 | 1.7 ± 0.3 | 4.4 ± 0.6 | 42.8 ± 8.8 | 217.4 ± 29.7 | 0.1 ± 0.1 |
| BDL 478 w4 | 1.7 ± 0.6 | 5.4 ± 1 | 47.8 ± 10 | 216.2 ± 26.1 | 0.3 ± 0.2 |
|  | | | | | |
| BDL 340 w8 | 0.9 ± 0.2 | 3.6 ± 0.8 | 41.7 ± 8 | 203 ± 25.5 | 0.3 ± 0.2 |
| BDL 468 w8 | 1.4 ± 0.4 | 4.8 ± 0.8 | 39.2 ± 7.8 | 199.2 ± 24.8 | 0.7 ± 0.4 |
| BDL 472 w7 | 1.5 ± 0.4 | 5.1 ± 1 | 39.3 ± 7.5 | 214.7 ± 25.2 | 0.6 ± 0.3 |

| ***Cerebellum*** | ***Number of*** ***microglia / 0.01 mm^2^*** | ***Number of processes*** | ***Processes length [µm]*** | ***Perimeter [µm]*** | ***Amoeboid microglia / 0.01 mm^2^*** |
| --- | --- | --- | --- | --- | --- |
| SHAM 388 | 1.1 ± 0.3 | 3.6 ± 0.9 | 35.9 ± 10.9 | 169 ± 20.2 | - |
| SHAM 469 | 0.9 ± 0.4 | 4.9 ± 0.7 | 38.3 ± 7.3 | 171.3 ± 11 | - |
| SHAM 470 | 1.2 ± 0.3 | 4.6 ± 0.6 | 38.5 ± 8.3 | 167.5 ± 22.4 | - |
|  | | | | | |
| BDL 475 w4 | 1.4 ± 0.5 | 3.8 ± 0.6 | 50.1± 9.5 | 213.8 ± 32.4 | 0.2 ± 0.2 |
| BDL 476 w4 | 1.3 ± 0.2 | 3.5 ± 0.5 | 52.8 ± 12.7 | 217.9 ± 28.5 | 0.3 ± 0.2 |
| BDL 478 w4 | 1.3 ± 0.3 | 3.7 ± 0.9 | 40.8 ± 7.4 | 166.7 ± 12.7 | 0.4 ± 0.2 |
|  | | | | | |
| BDL 340 w8 | 1.1 ± 0.5 | 5.3 ± 0.5 | 43 ± 8.6 | 205.5 ± 23.1 | 0.6 ± 0.3 |
| BDL 468 w8 | 1 ± 0.4 | 4.7 ± 0.7 | 40 ± 7.7 | 208.5 ± 25.6 | 0.7 ± 0.5 |
| BDL 472 w7 | 1.1 ± 0.2 | 3.9 ± 1.1 | 41.8 ± 8.7 | 203.6 ± 26.5 | 0.7 ± 0.2 |

| ***Striatum*** | ***Number of*** ***microglia / 0.01 mm^2^*** | ***Number of processes*** | ***Processes length [µm]*** | ***Perimeter [µm]*** | ***Amoeboid microglia / 0.01 mm^2^*** |
| --- | --- | --- | --- | --- | --- |
| SHAM 388 | 1.2 ± 0.4 | 4.8± 0.9 | 34.7± 7.9 | 175.8 ± 21.9 | - |
| SHAM 469 | 1.1 ± 0.4 | 5.2 ± 0.9 | 38.7 ± 7 | 182.7 ± 26.5 | - |
| SHAM 470 | 1.3 ± 0.2 | 5.3 ± 0.7 | 35.7 ± 7.5 | 188.8 ± 20.8 | - |
|  | | | | | |
| BDL 475 w4 | 1.7 ± 0.4 | 4.8 ± 1 | 44.8 ± 10.9 | 206.4 ± 24.7 | 0.1 ± 0.1 |
| BDL 476 w4 | 1.3 ± 0.3 | 4.3 ± 0.5 | 40.4 ± 3.2 | 200.8 ± 20.6 | 0.2 ± 0.2 |
| BDL 478 w4 | 1.9 ± 0.8 | 5.5 ± 1 | 38.9 ± 7.7 | 196.9 ± 13 | 0.5 ± 0.4 |
|  | | | | | |
| BDL 340 w8 | 1 ± 0.2 | 3.8 ± 0.9 | 37.8 ± 7 | 194.4 ± 23.6 | 0.4 ± 0.2 |
| BDL 468 w8 | 1.2 ± 0.2 | 4.9 ± 0.9 | 37.9 ± 7.8 | 190.6 ± 23.9 | 0.5 ± 0.5 |
| BDL 472 w7 | 1.7 ± 0.4 | 4.9 ± 1.2 | 38.3 ± 8.8 | 207.9 ± 25.8 | 1.2 ± 0.7 |

**Table S5. Results of the Golgi**-**Cox staining of hippocampal and cerebellar neurons.** Changes in neuronal soma and apical and basal dendrites length are presented as mean ± SD with the corresponding relative percentage changes. One-way Anova with post-hoc Tukey HSD, *p<0.05, **p<0.01, ***p<0.001, ****p<0.0001, mean ± SD.

| ***Hippocampus CA1*** | ***Neuronal soma [µm^2^]*** | ***Spines / 10μm dendrites length*** | |
| --- | --- | --- | --- |
|  |  | ***Apical*** | ***Basal*** |
| ***Sham*** | 227.47 ± 60.81 | 9.65 ± 2.09 | 9.97 ± 1.93 |
| ***BDL w8*** | 385.74 ± 105.43 | 6.41 ± 1.67 | 6.06 ± 1.49 |
| ***Relative change [%]*** | | | |
| ***Sham vs. BDL w8*** | +69.57*** | - 33.64*** | - 39.20*** |

| ***Hippocampus DG*** | ***Neuronal soma [µm^2^]*** | ***Apical Spines / 10μm dendrites length*** |
| --- | --- | --- |
| ***Sham*** | 137.50 ± 36.21 | 9.56 1.54 |
| ***BDL w8*** | 175.13 ± 49.07 | 6.45 ± 1.73 |
| ***Relative change [%]*** | | |
| ***Sham vs. BDL w8*** | + 27.36*** | - 32.51*** |

| ***Cerebellum - Purkinje Cells*** | ***Neuronal soma [µm^2^]*** | ***Apical Spines / 10μm dendrites length*** |
| --- | --- | --- |
| ***Sham*** | 488.28 ± 111.27 | 12.89 ± 2.14 |
| ***BDL w8*** | 347.42 ± 87.86 | 9.19 ± 1.81 |
| ***Relative change [%]*** | | |
| ***Sham vs. BDL w8*** | -28.84*** | - 28.67**** |

**Golgi** **–Cox:** mean ± SD per animal

|  | **CA1** | | | **DG** | | ***Purkinje Cells*** | |
| --- | --- | --- | --- | --- | --- | --- | --- |
|  | ***Neuronal soma [µm^2^]*** | ***Spines / 10μm dendrites length*** | | ***Neuronal soma [µm^2^]*** | ***Spines / 10μm dendrites length*** | ***Neuronal soma [µm^2^]*** | ***Spines / 10μm dendrites length*** |
|  |  | ***Apical*** | ***Basal*** |  | ***Apical*** |  | ***Apical*** |
| SHAM 358 | 211.4 +-47.4 | 10.5 +- 2.7 | 9.8 +- 2.4 | 131.3 +- 29.3 | 9.1 +- 1.8 | 539.2 +- 104.1 | 13 +- 2.2 |
| SHAM 359 | 228.9 +- 84.4 | 9.9 +- 1.1 | 10.4 +- 1.3 | 131.5 +- 40.9 | 10 +- 1.6 | 435.7 +- 88.7 | 12.4 +- 2.6 |
| SHAM 360 | 290.2 +- 34.8 | 8.3 +- 1.3 | 9.7 +- 2 | 116.7 +- 25.2 | 11.4 +- 2.4 | 495.7 +- 45.6 | 11.8 +- 1.7 |
| SHAM 361 | 232.6 +- 50.7 | 10.5 +- 2.1 | 10.1 +- 1.3 | 142.9 +- 35.9 | 10 +- 1.1 | 518.9 +- 85.3 | 12.6 +- 1.7 |
| SHAM 362 | 214.3 +- 61.8 | 9.61 +- 2 | 10 +- 2.4 | 157 +- 34.3 | 9.1 +- 1.4 | 431 +- 142.6 | 15 +- 1 |
| SHAM 363 | 222.3 +- 51.3 | 9.2 +- 2.3 | 9.6 +- 2.6 | 104.7 +- 13.3 | 7.1 +- 2 | 550.9 +-69.8 | 12.6 +- 1.4 |
|  | | | | | | | |
| BDL 355 | 453.8 +- 60.5 | 4 +- 0.9 | 4.1 +- 1.5 | 132.3 +- 18.4 | 5.1 +- 1.3 | 391.2 +- 108.9 | 9.6 +- 1.7 |
| BDL 356 | 429.5 +- 116.9 | 5.9 +- 1.5 | 5.1 +- 0.9 | 124.3 +- 17.4 | 4.8 +- 2.6 | 432.3 +- 74.2 | 9.1 +- 1.9 |
| BDL 357 | 427.9 +- 109.9 | 6.7 +- 1.5 | 6.4 +- 1.5 | 155 +- 41.6 | 6.7 +- 1.8 | 339.7 +- 76.1 | 9 +- 1.8 |
| BDL 365 | 358 +- 78.3 | 5.1 +- 1.2 | 6 +- 1 | 249.7 +- 21.3 | 5.9 +- 0.9 | 412.4 +- 71.9 | 8.9 +- 2.2 |
| BDL 367 | 303.4 +- 70.5 | 7.1 +- 1.6 | 6.5 +- 1.1 | 196.5 +-50 | 5.9 +- 1.8 | 296.1 +- 72.4 | 9 +- 1.6 |
| BDL 368 | 341.6 +- 69 | 6.9 +- 1.5 | 6.2 +- 1.7 | 181.6 +- 43.7 | 7 +- 1.3 | 346.5 +- 77.4 | 9.6 +- 1.9 |

**Fig. S8. Representative micrograph of DAPI stained brain sections illustrating type II astrocytes** (arrowheads). Type II astrocytes show nuclear enlargement, chromatin migration and clearing (scale bar = 25μm).

**Fig. S9. Representative micrographs of histological sections of ventricles.** Anti-Iba1 (green) and DAPI (blue) staining of **(A)** mesencephalic cerebral aqueduct and **(B)** telencephalic lateral ventricle. Activated macrophages accumulation and clot-like formation. like formation. White arrow - increased number of macrophages; white arrowhead – macrophages clot-like formation. Stitched reconstruction was generated only for the BDL samples, where an increased presence of macrophages was observed and therefore needed a broader field of view.

**Fig. S10. Representative micrographs of histological sections of Golgi-Cox staining and neuronal morphology analysis of hippocampal CA1 neurons.** One-way Anova with post-hoc Tukey HSD: *p<0.05, **p<0.01, ***p<0.001, ****p<0.0001, mean ± SD. A sub-group of rats (No. BDL 355, 356, 365, 368 and Sham 360, 363) used to create this figure have been previously described and characterized in earlier publications^4,6^.

**References**

1.	DeMorrow S, Cudalbu C, Davies N, Jayakumar AR, Rose CF. 2021 ISHEN guidelines on animal models of hepatic encephalopathy. *Liver International*. 2021;41(7):1474-1488. doi:10.1111/liv.14911

2.	Braissant O, Rackayová V, Pierzchala K, Grosse J, McLin VA, Cudalbu C. Longitudinal neurometabolic changes in the hippocampus of a rat model of chronic hepatic encephalopathy. *Journal of Hepatology*. 2019;71(3):505-515. doi:10.1016/j.jhep.2019.05.022

3.	Louth EL, Sutton CD, Mendell AL, MacLusky NJ, Bailey CDC. Imaging Neurons within Thick Brain Sections Using the Golgi-Cox Method. *J Vis Exp*. 2017;(122):55358. doi:10.3791/55358

4.	Mosso J, Briand G, Pierzchala K, et al. Diffusion of brain metabolites highlights altered brain microstructure in type C hepatic encephalopathy: a 9.4 T preliminary study. *Front Neurosci*. 2024;18:1344076. doi:10.3389/fnins.2024.1344076

5.	Zaqout S, Kaindl AM. Golgi-Cox Staining Step by Step. *Front Neuroanat*. 2016;10. doi:10.3389/fnana.2016.00038

6.	Giovannoni L, Pierzchala K, De Roo M, et al. Effects of cholestasis and hyperammonemia on dendritic spine density and turnover in rat hippocampal neurons. *Sci Rep*. 2024;14(1):29841. doi:10.1038/s41598-024-80871-8

7.	Račkayová V, Simicic D, Donati G, et al. Late post-natal neurometabolic development in healthy male rats using 1H and 31P magnetic resonance spectroscopy. *Journal of Neurochemistry*. 2021;157(3):508-519. doi:10.1111/jnc.15294

8.	Tkáč I, Rao R, Georgieff MK, Gruetter R. Developmental and regional changes in the neurochemical profile of the rat brain determined by in vivo 1H NMR spectroscopy. *Magnetic Resonance in Medicine*. 2003;50(1):24-32. doi:10.1002/mrm.10497

9.	RStudio Team. (2021). RStudio: Integrated Development Environment for R. RStudio, PBC. http://www.rstudio.com/

10.	Cudalbu C, Bady P, Lai M, et al. Metabolic and transcriptomic profiles of glioblastoma invasion revealed by comparisons between patients and corresponding orthotopic xenografts in mice. *acta neuropathol commun*. 2021;9(1):133. doi:10.1186/s40478-021-01232-4

11.	Lai M, Vassallo I, Lanz B, et al. In vivo characterization of brain metabolism by 1H MRS, 13C MRS and 18FDG PET reveals significant glucose oxidation of invasively growing glioma cells. *International Journal of Cancer*. 2018;143(1):127-138. doi:10.1002/ijc.31299

12.	Abdi H, Williams LJ, Valentin D, Bennani-Dosse M. STATIS and DISTATIS: optimum multitable principal component analysis and three way metric multidimensional scaling. *WIREs Computational Statistics*. 2012;4(2):124-167. doi:10.1002/wics.198

13.	Lavit C, Escoufier Y, Sabatier R, Traissac P. The ACT (STATIS method). *Computational Statistics & Data Analysis*. 1994;18(1):97-119. doi:10.1016/0167-9473(94)90134-1
